# Crystal structure of a cytocidal protein from lamprey and its mechanism of action in the selective killing of cancer cells

**DOI:** 10.1186/s12964-019-0358-y

**Published:** 2019-05-27

**Authors:** Yue Pang, Meng Gou, Kai Yang, Jiali Lu, Yinglun Han, Hongming Teng, Changzhi Li, Haina Wang, Caigang Liu, Kejia Zhang, Yongliang Yang, Qingwei Li

**Affiliations:** 1 College of Life Science, Liaoning Normal University, Dalian, 116081 China; 2 Lamprey Research Center, Liaoning Normal University, Dalian, 116081 China; 3 Center for Molecular Medicine, School of Life Science and Biotechnology, Dalian University of Technology, Dalian, 116023 China; 4 Department of Breast Surgery, Shengjing Hospital of China Medical University, Shenyang, 110004 China

**Keywords:** Lamprey, LIP, Cytotoxic activity, Selective recognition, GPI-APs

## Abstract

**Background:**

In previous research, we found that lamprey immune protein (LIP) possessed cytocidal activity against tumor cells, but the mechanism of the selective recognition and killing of tumor cells by LIP was not identified.

**Methods:**

Superresolution microscopy, crystallographic structural analysis, glycan chip assay, SPR experiments, FACS assays, computational studies and mass spectrometric analysis firmly establish the mode of action of LIP, which involves dual selective recognition and efficient binding.

**Results:**

We determined the overall crystallographic structure of LIP at a resolution of 2.25 Å. LIP exhibits an elongated structure with dimensions of 105 Å × 30 Å × 30 Å containing an N-terminal lectin module and a C-terminal aerolysin module. Moreover, the Phe^209^-Gly^232^ region is predicted to insert into the lipid bilayer to form a transmembrane β-barrel, in which the hydrophobic residues face the lipid bilayer, and the polar residues constitute the hydrophilic lumen of the pore. We found that LIP is able to kill various human cancer cells with minimal effects on normal cells. Notably, by coupling biochemical and computational studies, we propose a hypothetical mechanism that involves dual selective recognition and efficient binding dependent on both N-linked glycans on GPI-anchored proteins (GPI-APs) and sphingomyelin (SM) in lipid rafts. Furthermore, specific binding of the lectin module with biantennary bisialylated nonfucosylated N-glycan or sialyl Lewis X-containing glycan structures on GPI-APs triggers substantial conformational changes in the aerolysin module, which interacts with SM, ultimately resulting in the formation of a membrane-bound oligomer in lipid rafts.

**Conclusions:**

LIP holds great potential for the application of a marine protein towards targeted cancer therapy and early diagnosis in humans.

**Electronic supplementary material:**

The online version of this article (10.1186/s12964-019-0358-y) contains supplementary material, which is available to authorized users.

## Background

Pore-forming toxins (PFTs) and pore-forming proteins (PFPs) have been identified in various organisms, including bacteria and eukaryotes [1]. Examples of well-characterized PFTs in eukaryotes include enterolobin from the Brazilian tree *Enterolobium contortisiliquum* [2], lectin from the mushroom *Laetiporus sulphureus* [3], amyloid-forming proteins [4], biomphalysin from the snail *Biomphalaria glabrata* [5, 6], the mammalian PFP perforin-2 [7, 8], human apoptosis-related Bcl2-like proteins [9], and the MAC/perforin-like protein complement component 9 [10]. The activities and structures of several new members of the aerolysin family have been characterized in eukaryotes; for instance, lysenin from the earthworm *Eisenia fetida* can specifically interact with sphingomyelin (SM) and may confer innate immunity against parasites by attacking the membranes of the parasites to form pores [11]. On the other hand, the βγ-CAT protein from the frog *Bombina maxima* counters microbial infection by eliciting rapid and effective host innate immune responses [12]. In zebrafish, it has been shown that specific binding of the lectin module of Dln1 to high-mannose glycans triggers substantial pH-dependent conformational changes in the aerolysin module, ultimately resulting in the formation of a membrane-bound octameric pore [13]. Recently, the pore-forming activity and structure of the human gasdermin D protein, which is involved in pyroptosis and inflammation, were determined [14, 15]. Accumulating evidence suggests that in addition to their ability to form channels in target cell membranes, eukaryotic PFPs can also serve as defense effectors to directly kill invading pathogens [6, 12, 14, 16] or assist hosts in prey disintegration, as in the case of hydralysins [17].

PFTs or PFPs mediate actions through cell surface receptors. Interestingly, some receptors appear to be unrelated except for the notable feature that they are all attached to cell surfaces via C-terminal glycosylphosphatidylinositol anchors (GPI-anchors) containing a glycan core and other sugars that may be added to the glycan core [18–21]. For example, an N-glycan on the GPI anchor is required by the channel-forming toxin aerolysin [18, 21]. However, it has been reported that PFPs can also bind membrane lipids, phosphoinositides [22], cardiolipin [14] and SM [23], with engagement of lipid- or N-glycan-binding proteins for recognizing the target membrane. PFPs oligomerize on the surface of the bilayer and are subsequently inserted into the bilayer to form a lesion. In this process, the PFPs undergo structural rearrangement to transition from a soluble state to a membrane-inserted state [24, 25].

In our previous research, a novel lamprey immune protein (LIP) from the supraneural body was purified and identified for the first time. LIP exhibits strong cytocidal activities against human tumor cells, with markedly divergent target cell specificities [26, 27]. LIP induces remarkable morphological changes in tumor cells, including cell blebbing, cytoskeletal alterations, mitochondrial fragmentation and endoplasmic reticulum vacuolation, and most of the cytoplasmic and organelle proteins are released following treatment with LIP. Our results regarding the antitumor potential of LIP in vivo showed that the injection of LIP into mice with tumors resulted in a vast amount of LIP localized on tumor cells, as well as the recruitment of macrophages, which directly induce tumor cell death. Moreover, slight morphological changes were observed in the endothelial cells of blood vessels, fibroblastic cells, and infiltrating inflammatory cells in the same tissue, even after LIP treatment [26]. The observations of mortality, body weight, and histopathology support the conclusion that LIP is safe for animals. However, the mechanism of the selective recognition and killing of cancer cells by LIP remains unknown. Here, we report the crystal structure of LIP containing an N-terminal jacalin-like domain and a C-terminal aerolysin domain. We found that LIP specifically recognizes not only biantennary, bisialylated, nonfucosylated N-glycan or sialyl Lewis X-containing glycan structures but also SM on lipid rafts. Our results suggest that LIP first binds with N-linked glycans on GPI-APs of tumor cells, undergoes a conformational change to facilitate binding of the C-terminal aerolysin domain with the SM of lipid rafts, and then facilitates the recruitment of additional LIP monomers to form polymers on the cell membrane. These findings suggest that LIP holds great potential for the application of a marine protein towards targeted cancer therapy in humans.

## Methods

### 3D-SIM superresolution microscopy

Cells were plated onto confocal dishes (coverglass-bottom dishes) and stained with Hoechst (Sigma) for 20 min to visualize the cell nuclei. Then, the cells were washed twice with PBS and labeled with Alexa Fluor 555-labeled cholera toxin B subunit (Molecular Probes, Invitrogen Corporation, Carlsbad, CA) for 2 min. The samples were labeled with Alexa 488-tagged LIP after washing with PBS and analyzed by 3D-SIM superresolution microscopy. The 3D-SIM images of the cells were acquired on a DeltaVision OMX V4 imaging system (GE Healthcare).

### Protein purification, crystallization and data collection

LIP was inserted into the expression vector pET28a at the TEV protease restriction site. The protein was purified with Ni beads. After removal of the His tag by overnight proteolysis at 4 °C, the tag-free LIP was purified by gel-filtration chromatography and concentrated to 17 mg/mL with 25 mM Tris-HCl and 150 mM NaCl (pH 7.5). The initial hits were observed upon crystallization in 2% 1,4-dioxane, 10% w/v polyethylene glycol 20,000, and 0.1 M bicine (pH 9.0) by the sitting drop method after 5 days. The crystals were flash-cooled at 100 K using 20% glycerol as an added cryoprotectant. The resolution of the best native data was 2.25 Å, and the space group was P4_3_2_1_2, as determined upon processing by the HKL2000 software.

### Structure determination and refinement

The structures were solved by molecular replacement with the Phenix software using a modified version of the structure 4ZNO. The initial phases were improved by rigid body refinement followed by rounds of simulated annealing refinement using the Phenix suite. Model rebuilding was performed manually with COOT. The final structure was refined to 2.25 Å by Refmac5 from CCP4. The data collection and structure refinement statistics are summarized in the data table. All figures representing structures were prepared with PyMOL.

### Glycan chip assay

A 100 N-glycan array on 8 subarray formats was used. Subarrays were assayed with glycan-binding protein biotinylated with N-hydroxysuccinimide (NHS)-biotin followed by a streptavidin-Cy5 conjugate. The array was scanned with a LuxScan 10 K microarray scanner at 475 PMT and 100% laser power at a wavelength of 635 nm. There was no nonspecific binding at the negative control spots. The positive control and marker exhibited binding as expected.

### Surface plasmon resonance (SPR) experiments

All surface plasmon resonance (SPR) experiments were performed in PBS (pH 7.4) at 25 °C using a BIAcore T200 instrument (GE Healthcare) at a flow rate of 30 μL/min. To measure the affinities of N025G and N003G (Chemily Glycoscience), the recombinant LIP was immobilized on a CM5 sensor chip (GE Healthcare) using an Amine Coupling Kit (GE Healthcare), resulting in a surface density of approximately 8000 resonance units (RU). For LIP to SM (Sigma S0756) kinetic assays, approximately 6000 RU of LIP was immobilized on a CM5 sensor chip, and regeneration was achieved with 10 mM glycine-HCl (pH 1.5). The binding kinetics were analyzed with BIAevaluation software, version 3.0, using the 1:1 binding model.

### Sucrose gradient fractionation and immunoblotting

Cells were grown to confluence (∼3 × 10^8^ cells) in medium supplemented with 10% normal human serum and then rinsed with PBS. After centrifugation, the cells were subjected to lipid extraction. The sucrose gradient fractionation method was as described in the literature [28]. After sucrose gradient fractionation, the samples were dot-blotted onto PVDF membranes. Membranes were blocked by incubation in a solution of powdered skim milk and then incubated with mouse monoclonal anti-flotillin-I in PBS with Tween 20 (PBST) for 4 h. The membrane was then washed with PBST and incubated for 45 min with the secondary antibody (HRP-conjugated anti-mouse IgG) in PBST. Blots were visualized using a western blotting detection system.

### Triton X-114 phase separation and N-glycosidase F treatment

The methods used have been previously described in the literature [28]. The sample was incubated with 0.5 U/mL N-glycosidase F for 30 h under constant stirring at 37 °C. The lipid was then centrifuged at 15000×g for 5 min, and the supernatant was analyzed by ligand fishing experiment on Biacore T200.

### Nano-LC-Q-TOF MS analysis

The mixtures were collected and transferred into new tubes for further Michrom AdvanceTM nano/cap LC-Q-TOF MS (Bruker, USA) analysis. Samples were loaded onto a trap column at 10000 nL/min, while chromatographic separation was performed at 200 nL/min. Mobile phases A and C consisted of 0.1% (v/v) formic acid in water, while mobile phase B consisted of 0.1% (v/v) formic acid in acetonitrile, and the gradient conditions were as follows: 5 to 40% B in 40 min and then up to 80% B in 4 min, maintaining this concentration for 10 min. The eluted glycans were directly introduced into a CaptiveSpray ionization-Q-TOF MS (Bruker, USA) for analysis. The dry temperature was set to 165 °C, and the capillary voltage was set to 1500 V. MS1 spectra were acquired from 50 to 2200 m/z at a resolution of approximately 30,000 (for m/z 445.1200).

### Data analysis

All MS/MS data were analyzed using Compass Data Analysis software 4.1 (Bruker, USA) and Proteinscape 3.0 with a glycan search engine (Bruker, USA). GlycomeDB (https://glytoucan.org) was used to identify glycans. The search parameters were as follows: Charge: 1+, 2+, 3+ and 4+; Taxonomy: *Homo sapiens*; Reducing end: 2AB; H+ up to 5, Na + up to 1 and K+ up to 1; MS tolerance: 0.05 Da; MS/MS Tolerance: 0.1 Da; Score > 20.0; fragmentation coverage> 15.0%; and intensity coverage> 15.0%.

### Recovery of LIP-bound ingredients

All experiments were carried out using a BIAcore T200 SPR sensor (GE Healthcare, USA) with BIAevaluation version 3.0 software and a CM5 sensor chip (carboxymethylated dextran surface). All assays were carried out at 25 °C. LIP was immobilized via amine groups in all four available flow cells. To this end, the chip surface was first activated by following a standard 1-ethyl-3-(3-dimethylaminopropyl)-carbodiimide (EDC)/NHS protocol, with BIAcore HBS-EP buffer used as the running buffer. LIP at a concentration of 0.2 mg/mL in 10 mM phosphate buffer (pH 7.4) was then injected for 10 min, which was followed by a 7 min injection of 1 M ethanolamine to inactivate the residual active groups. Typically, approximately 15,000 RU of LIP was immobilized per flow cell. The sample was diluted sixfold in HBS-N buffer, and approximately 3800 μL was injected at a flow rate of 30 μL/min. All four flow cells were used for analyte injection, and 30 times the injected volume was recovered. The flow system was then washed with 0.5% TFA and rinsed with 50 mM NH_4_HCO_3_, and the flow cells were rinsed with 50 mM NH_4_HCO_3_. The bound material was eluted with 100 mL of 0.5% TFA and 50 mM NH_4_HCO_3_ for analysis of the trypsin digest. HBS-N buffer was used as the running buffer.

### Treatments prior to LIP exposure

Cell surface SM was depleted with SMase in RPMI 1640 without fetal calf serum (FCS) at 37 °C for 30 min. PI-PLC (Sigma P5542), which cleaves the phosphoglycerol bond found in GPI-APs, was used to release GPI-linked proteins from the outer cell membrane. Cells treated with PI-PLC (5 U/mL) in RPMI 1640 without FCS for 1 h at 37 °C were incubated with LIP (4 μg/mL) or Alexa488-tagged LIP for various durations.

### MALDI-TOF-MS analysis of sphingomyelin (SM)

Samples were analyzed with a MALDI-TOF mass spectrometer in positive ion mode. For MS analysis, the dried sample was resuspended in 10 μL of methanol/trichloromethane (1:1, v/v). A total of 0.5 μL of matrix solution (10 mg of 2,5-DHB dissolved in 1 mL of 30% ethanol) and 0.5 μL of the diluted analyte solution were spotted on the MALDI target plate (Bruker Daltonics). Then, the plate was analyzed by an ultrafleXtreme mass spectrometer (Bruker Corporation, Germany), which was controlled by flexControl 3.4 software (build 119) (Bruker Daltonics).

### Preparation of artificial membrane liposomes and calcein leakage experiments

Artificial membrane liposomes were prepared as reported previously [29, 30]. Liposomes comprising DOPC and SM at a ratio of 3:7 or 7:3 (by weight) were prepared. Liposomes and LIP (or control protein, 10% Triton X-100) were added to black 96-well microtiter plates, and fluorescence values were measured at different times using a Thermo Scientific Varioskan Flash instrument (Thermo scientific, USA). The reaction buffer self-quenched its fluorescence, resulting in low background fluorescence intensity of the vesicle dispersion (Fo). The release of calcein, which was caused by LIP addition, led to dilution of the dye in the medium, and the release of the dye could then be monitored as an increase in fluorescence intensity (Ft). The experiments were normalized to the total fluorescence intensity (Fmax), which corresponded to the total dye released after complete disruption of all the vesicles by Triton X-100 (0.1%). The results of the leakage experiments are presented as percent released calcein, which was calculated as follows: RF(%) = 100(Ft-Fo)/(Fmax-Fo). The calcein fluorescence excitation and emission wavelengths were 470 nm and 520 nm, respectively.

### Cell membrane SM assay

SM is mainly found in the exoplasmic leaflet of the cell membrane. SM activity was analyzed using the Sphingomyelin Assay Kit (ab138877; Abcam, USA) with the AbRed indicator as a fluorogenic probe to indirectly quantify the phosphocholine produced by the hydrolysis of SM by SMase. Briefly, 5 × 10^6^ cells were harvested and lysed and were then used to measure the SM content using the Sphingomyelin Assay Kit (Fluorometric) according to the manufacturer’s specifications. After 1 h of incubation at room temperature in the dark, the microtiter plate was read using a fluorescence microplate reader with excitation and emission wavelengths of 540 and 590 nm, respectively. A standard curve was prepared by serial dilutions (from 0.1 to 100 μM) of a 50 mM SM standard stock solution. For each sample, SM levels were calculated from the difference in fluorescence between the sample and the corresponding negative control. The experiments were performed in triplicate.

### Real-time PCR

Total RNA from cancer and normal cells was extracted using TRIzol (Life Technologies) and treated with DNase I (TaKaRa, China). Control reactions lacking reverse transcriptase (No-RT) were prepared for each sample. Reverse transcription was performed on 2 μg total RNA using oligo (dT) and the PrimeScript™ RT Reagent Kit with gDNA Eraser Mix according to the supplier’s instructions (TaKaRa). Quantitative PCR was performed with SYBR Green Premix Ex Taq (TaKaRa) using an Applied Biosystems 7500 Fast real-time PCR system (Life Technologies). Each sample was run in triplicate. The relative cDNA level was calculated by the comparative CT (cycle threshold) method and normalized to an internal control, glyceraldehyde 3-phosphate dehydrogenase (*gapdh*). PCR primers included (5′ to 3′):

- h-*SMS1* sense (CTGGTTAATTCAGTGGCTGCTCTT),
- h-*SMS1* antisense (TTCGGAGAACAGTTGAAATGCA),
- h-*SMS2* sense (CCTGTGCCTGGAATGCATTT),
- h-*SMS2* antisense (GTGACCGCTGAAGAGGAAGTCT),
- h-*Sphk1* sense (ATGCTGGCTATGAGCAGGTC),
- h-*Sphk1* antisense (ACATCAGCAATGAAGCCCCA),
- h-*Gapdh* sense (TGACGCTGGGGCTGGCATTG),
- h-*Gapdh* antisense (GGCTGGTGGTCCAGGGGTCT).

The specificity of qPCR was validated by melting curve analysis. Data are shown as the means ± SD from three independent experiments, and *p*-values were calculated using Student’s t-test (*P < 0.05, **P< 0.01, ***P < 0.001).

### Computational modeling and molecular dynamics (MD) simulation

The monomeric structure of LIP was used for all the simulations. Notably, only the aerolysin module was used in the simulation of LIP complexed with SM to reduce the computational cost. Molecular docking was performed using our newly developed in-house docking tool FIPSDock [31]. The ligand and protein input structures in the simulations were saved using PDBQT file format. All of the MD simulations were performed by Gromacs 4.6.7 with the Amber99sb force field. The general amber force field (GAFF) parameters for all the disaccharides and SM were built by UCSF Chimera and ACPYPE with AM1-BCC charges for the small molecules. The protocol of the MD simulation was as follows: 1) The complex structure was solvated in a truncated octahedron TIP3P water box with a 1-nm distance from the edge and relaxed using 1000 steps of steep descent minimization followed by 5000 steps of conjugate gradient minimization. 2) The complex was then equilibrated under standard NVT conditions for 1 ns. 3) After the equilibration run, a 5-ns simulation at constant pressure with a target temperature of 300 K and pressure of 1 atm was conducted. The particle mesh Ewald (PME) method implemented in Gromacs 4.6.7 was used to treat the long-range electrostatic interactions in the production phase. The LINCS algorithm was employed to restrain the hydrogen positions at their equilibrium distances. 4) Both the energies and coordinates were saved every 10 ps for the postproduction analysis. After MD simulation, the molecular mechanics energies combined with the Poisson–Boltzmann surface area continuum solvation (MM/PBSA) method were employed to estimate the free energy of the binding of disaccharides and sphingomyelin (SM) with LIP protein.

### Steady-state fluorescence spectroscopy studies

Steady-state fluorescence spectra were obtained with a PerkinElmer PE LS-55 luminescence/fluorescence spectrophotometer. The excitation wavelength was 280 nm, and the emission spectra were obtained at wavelengths ranging from 290 to 495 nm. The excitation and emission slits were fixed at 10.0 and 5.0 nm, respectively. The temperature was set at 25 °C. To make the LIP-N003G or LIP-SM complex, the LIP stock solutions and N003G or SM stock solutions, respectively, were mixed in phosphate buffer. The resultant mixture was equilibrated for 2 min before recording the steady-state fluorescence spectrum.

### Stopped-flow measurements

The stopped-flow experiments were conducted on an Applied Photophysics Model SX20 stopped-flow spectrofluorimeter fitted with a xenon lamp. All reactions were performed in 100 mM potassium phosphate buffer (pH 7.0) at 25 °C with an LIP concentration of 5.88 μM and an N003G concentration of 600 μM. A volume of 50 μL was injected from each syringe each time, and the reported concentrations are the concentration observed in the reaction chamber. The fluorescence emission intensity was monitored at wavelengths above 310 nm using a 305-nm cut-off filter with an excitation wavelength of 280 nm and a slit width of 1 nm.

### LDH efflux measurements

For determination of the LDH efflux from the cells, the medium was centrifuged to remove floating cells. Next, the supernatant was mixed with the solution of the LDH cytotoxicity detection kit (Takara), and the optical densities at 490 nm were measured with a microplate reader model 550 (Bio-Rad). The amounts of leaked LDH were determined and represented as percentages of the LDH activity obtained after treatment of the cells with 1% (w/v) Triton X-100.

### Statistical analysis

All statistical analyses were done using GraphPad Prism 5.0 software. Differences between treatment groups were determined by Student’s t-test. *P* < 0.05 was set as the threshold for significance (*P<0.05,  **P < 0.01). Bar charts show the means ± SDs of three independent experiments.

## Results

### Selective killing of tumor cells by recombinant LIP

**Fig. 1:**
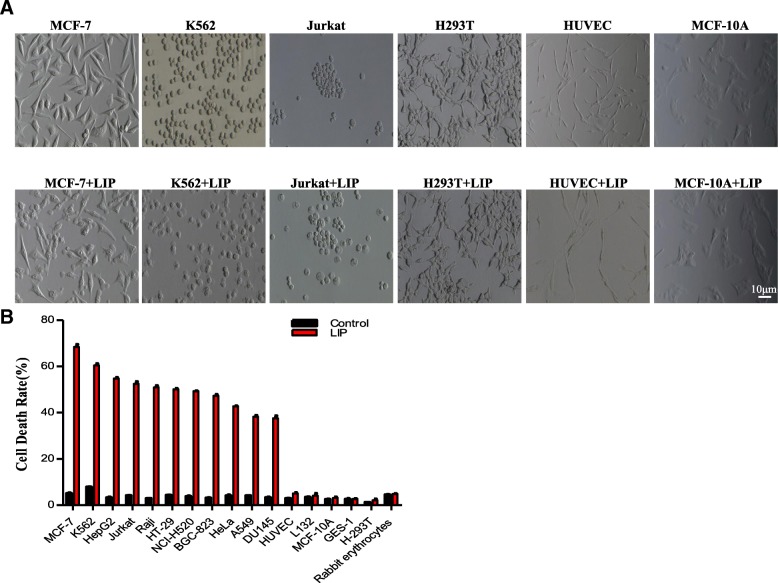
Selective cytocidal activity of LIP in vitro. (**a**) Morphological alterations of cells induced by LIP. The cells were incubated with 1 μg/mL LIP at 37 °C for 24 h, observed with a phase-contrast microscope and photographed. (**b**) Cytocidal activity of LIP against cultured cancer cell lines and normal cells. A total of 5 × 10^4^ cells were preincubated at 37 °C for 20 h and then treated with LIP (final concentration, 1 μg/mL) for 24 h at 37 °C. The cytotoxic activity of LIP was determined using the LDH Cytotoxicity Detection Kit. Each histogram represents the average value of triplicate experiments. Means ± SDs are shown

Our previous study showed that cell secretions from adult lamprey supraneural body tissues exhibit cytocidal activity against tumor cells [32]. Subsequently, we purified and identified a novel protein, LIP, as a candidate factor for such actions [26]. The effects of LIP on several types of cancer cells were evaluated, showing the apparent induction of cell swelling and bursting of target cells (Fig. 1a). The killing efficacy of LIP against various cultured human cancer cells and normal cells was also studied. LIP was found to have consistent cytocidal activity against various human cancer cells at a dose of 1 μg/mL (Fig. 1b). For example, LIP exhibited high cytotoxicity towards human breast carcinoma cells (MCF-7 cells) and low cytotoxicity towards normal breast cells (MCF-10A cells). LIP markedly induced cell death in cancer cells in a dose-dependent manner (see the tested cancer cell lines listed in Additional file 2: Table S1 and the tested normal and primary cell lines listed in Additional file 2: Table S2). A total of 23 cancer cell lines were screened, and all showed more than a 30% decrease in viability 24 h after exposure to LIP. In contrast, LIP exposure had barely any effect on the viability of normal cells, even after prolonged exposure and high doses.

### Oligomerization of LIP localized in lipid rafts of cancer cells

**Fig. 2:**
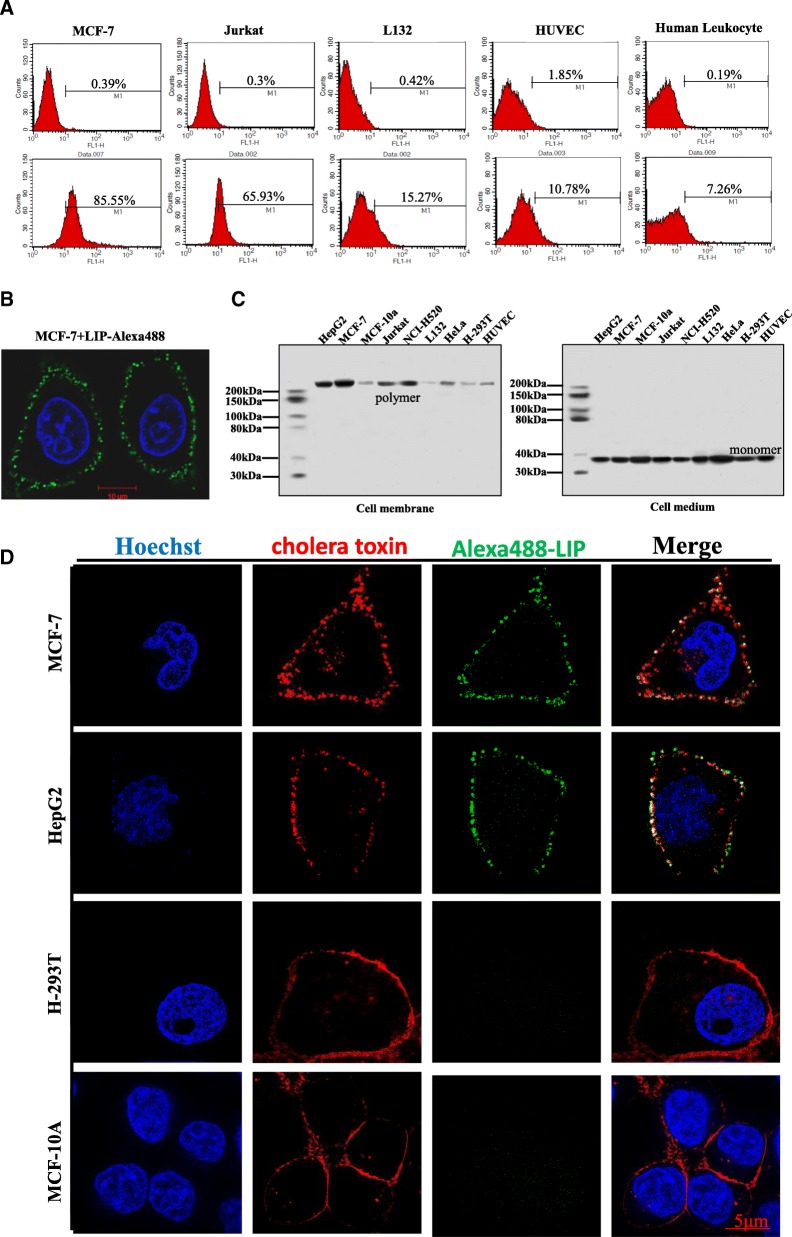
Localization of LIP in the lipid raft microdomains of cancer cell membranes. (**a**) A total of 5 × 10^4^ cancer cells or normal cells were incubated with Alexa488-tagged LIP (1 μg/mL) at 37 °C for 30 min and then subjected to flow cytometric analysis. The upper panels and lower panels show the results before and after LIP treatment, respectively. (**b**) The cells were observed and photographed using a Zeiss LSM 780 inverted microscope (magnification: 63×). (**c**) The cells were incubated with LIP (1 μg/mL) at 37 °C for 30 min. The cell membranes and culture medium were independently collected, resolved by SDS-PAGE and probed by western blotting using anti-LIP antibodies. (**d**) MCF-7, HepG2, H293T and MCF-10A cells were stained with Alexa555-cholera toxin subunit B (CT-B) prior to staining with Alexa488-tagged LIP. The CT-B was used following the instructions from Thermo Fisher Scientific. The cells were observed and photographed by 3D-SIM superresolution microscopy

Next, we examined the localization of LIP on cancer cells. Flow cytometry with Alexa488-labeled LIP showed that LIP bound to the target cancer cells and was associated with the plasma membrane until the cells were damaged (Fig. 2a, b). Moreover, the amount of LIP localized on the cancer cells was higher than that on normal cells and correlated with the cell death rate (Fig. 2a and Additional file 1: Figure S1). Western blotting analysis indicated that the LIP on cell membranes was in the form of a polymer, while LIP in the culture medium was a monomer (Fig. 2c), suggesting that the binding of LIP to cancer cells triggered the oligomerization process. Intriguingly, the LIP oligomers were not detected in normal cells. Next, we examined whether lipid rafts play a role in the induction of cancer cell death by LIP and found that the Alexa488-labeled LIP was colocalized with the lipid raft marker, namely, cholera toxin subunit in cancer cells, but little or no Alexa488-labeled LIP was detected on the lipid rafts of normal cells (Fig. 2d).

### Determination of the LIP structure

**Fig. 3:**
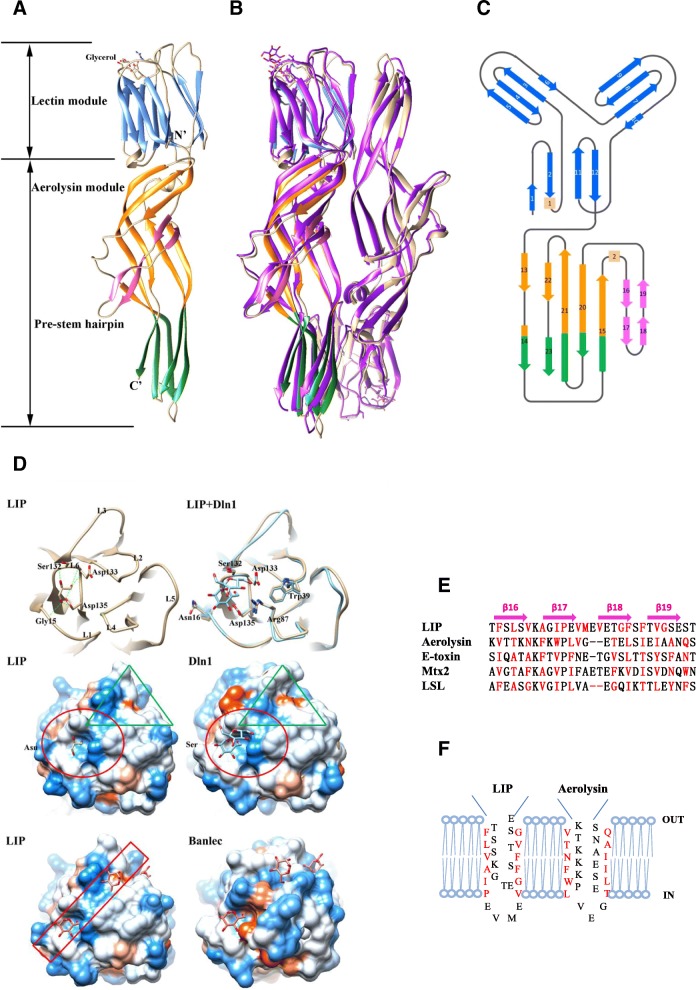
Overall structure of the LIP dimer. (**a**) Overall structure of LIP. The lectin module and the middle and C-terminal moieties of the aerolysin module of the LIP subunit are shown in blue, orange, and green, respectively. The prestem hairpin (the putative transmembrane region) is shown in pink. The N and C termini as well as the bound glycerol are labeled. (**b**) Superimposition of LIP with Dln1. The second subunit of LIP in the asymmetric unit is shown in sandy brown, and Dln1 is shown in purple. (**c**) Topology diagram of the LIP monomer. (**d**) Comparison of the ligand-binding sites of LIP (sandy brown) and Dln1 (blue). The interactions of the bound glycerol molecule and residues Gly^15^, Ser^132^, Asp^133^ and Asp^135^ of the lectin module in LIP are presented (upper panel). The lectin modules of LIP and Dln1 are superimposed. The interactions of the bound sucrose and the residues in the pocket in Dln1 are labeled, as are the corresponding residues in LIP (upper panel). The hydrophobicity of the surfaces of LIP and Dln1 is depicted according to the Kyte-Doolittle scale with colors ranging from Dodger blue for the most hydrophilic to white at 0.0 and orange-red for the most hydrophobic [33] (middle panel). The red circles are the ligand-binding sites. These sites are almost identical, except for the part on the left, which is an asparagine (Asn) in LIP but a serine (Ser) in Dln1. The green triangle is the channel extending from the binding site. Surface representations of LIP and Banlec (lower panel). Banlec and LIP are superimposed, and the ligands from Banlec are presented with the surface of LIP. The belt-shaped channel is marked with a red rectangle. (**e**) Multiple-sequence alignment of the putative transmembrane region from different aerolysin members. The members include LIP (*Lampetra japonica*), aerolysin (*Aeromonas sobria*), E-toxin (*Clostridium perfringens*), Mtx2 (*Lysinibacillus sphaericus*) and LSL (*Laetiporus sulphureus*). Alignments were generated based on the alternating patterns of polar and hydrophobic residues. The hydrophilic residues (facing the pore lumen) and hydrophobic residues (facing the lipid bilayer) are marked in black and red, respectively. (**f**) Schematic representation of the antiparallel strands forming the β-barrel of LIP and the corresponding residues of aerolysin. The alignment is based on previous reports [21] and sequence similarity. The residues are depicted either facing the lipid bilayer or lining the lumen of the pore

In the present study, the three-dimensional structure of LIP was determined. It belonged to the P4_3_2_1_2 space group with an antiparallel homodimer in the asymmetric unit (Fig. 3a and b). The model exhibited good stereochemistry and the quality of the final model was assessed by the statistics given in Additional file 2: Table S3. Similar to the zebrafish aerolysin-like protein Dln1 [13] previously described (Fig. 3b), the interface between the two antiparallel subunits was mainly stabilized by salt bridges and hydrogen bonds. Each subunit displayed an elongated structure with dimensions of 105 Å × 30 Å × 30 Å consisting of an N-terminal lectin module and a C-terminal aerolysin module (Fig. 3c). Notably, β-strands were dominant in both modules.

### The lectin module of LIP

The N-terminal lectin module of LIP shared the highest structural similarity with mannose-specific jacalin-related lectins (mJRLs) [34], such as the lectin Banlec (PDB 3MIT) from the banana species *Musa paradisiac*, the *Helianthus tuberosus* lectin Heltuba (PDB 1C3K), the antiviral lectin griffithsin (GRFT) (PDB 3LL0) from the red alga *Griffithsia* sp., the human pancreatic lectin ZG16p (PDB 3VY7), and the lectin module of zebrafish Dln1 (PDB 4ZNO), with a Z-score of 17–29 and a root-mean-square deviation (RMSD) of 2.2–0.7 Å over ~ 123 Cα atoms (Additional file 2: Table S4). Interestingly, despite the structural similarities, these lectins shared low sequence homology, with identities ranging from 20 to 40% (Additional File 1: Figure S2A). The lectin module of LIP adopted a conserved three-sided β-prism conformation with three Greek key motifs consisting of 4-stranded β-sheets. Consistent with the lectin structures [35], the putative primary sugar-binding site of the lectin module in LIP may consist of a GG loop (L1), a ligand-binding loop (L6) and a ligand recognition loop (L4) (Additional File 1: Figure S2B). The residues Ser132, Asp133, and Asp135 in the binding loop and Gly15 in the GG loop enable a hydrogen bond network with glycerol, which was used as the cryoprotectant (Fig. 3d). In the crystal structure, this ligand binding site is most likely partially occupied by one glycerol. A second potential ligand-binding site, also observed in Banlec [35, 36], involved L2 (ligand-binding loop) and L3 (GG loop) and may play an important role in binding carbohydrate and making numerous interactions with the protein. Upon superimposing the structure of the lectin module of LIP and Dln1, similar conformation of binding pocket residue Trp39, Arg87, Ser132, Asp133 and Asp135 were observed. The bound glycerol molecule was stabilized by the hydrogen bond network between these residues (Fig. 3d). Besides, a substitution of Ser with Asn at position 16 in LIP was presented, which may also be involved in ligand binding. The structure of the channel extending from the binding site was also different. The channel in LIP was relatively narrower and deeper compared to that in Dln1(Fig. 3d).

### The aerolysin module of LIP

The C-terminal module was named the aerolysin module owing to the structural similarity to aerolysin family proteins, such as the *A. hydrophila* aerolysin (PDB 1PRE), the *L. sulphureus* lectin LSL (PDB 1W3A), and the aerolysin module of zebrafish Dln1 (PDB 4ZNO), with Z-scores ranging from 7 to 22 and RMSDs of 4.7–1.1 Å over ~ 147 Cα atoms (Additional file 2: Table. S4). The aerolysin module could be divided into two segments (Fig. 3c). The segment at the central moiety consisted of an amphipathic hairpin covering a twisted antiparallel five-stranded β-sheet, expected to form a β-barrel structure, which is conserved in aerolysin family proteins. In the other segment, i.e., the C-terminal segment, the five strands were arranged in a two-stranded β-sheet and a three-stranded β-sheet, packing against each other to form a distinctive β-sandwich. The long antiparallel pair of β-strands turns at the distal end of the C-terminal segment with a pair of short loops. The C-terminus β-sheet residue is located near the end of the antiparallel pair of β-strands with the tail extending outward. Interestingly, despite the 50–60% identities of LIP with bacterial PFTs (e.g. *A. hydrophila* aerolysin, E-toxin, Mtx2 and LSL), patterns of amino acid arrangement in the pre-stem hairpins exhibit significant conservation (Fig. 3e). A previous report demonstrated that *Clostridium perfringens* enterotoxin (CPE) region was involved in the formation of pores after the assembly of a pre-pore oligomeric complex [37], which was believed to be inserted into the lipid bilayer to form a transmembrane β-barrel where the hydrophobic residues faced the lipid bilayer, and the polar residues constituted the hydrophilic lumen of the pore (Fig. 3f).

### Sialylated antennary N-glycan specificity of LIP

**Fig. 4:**
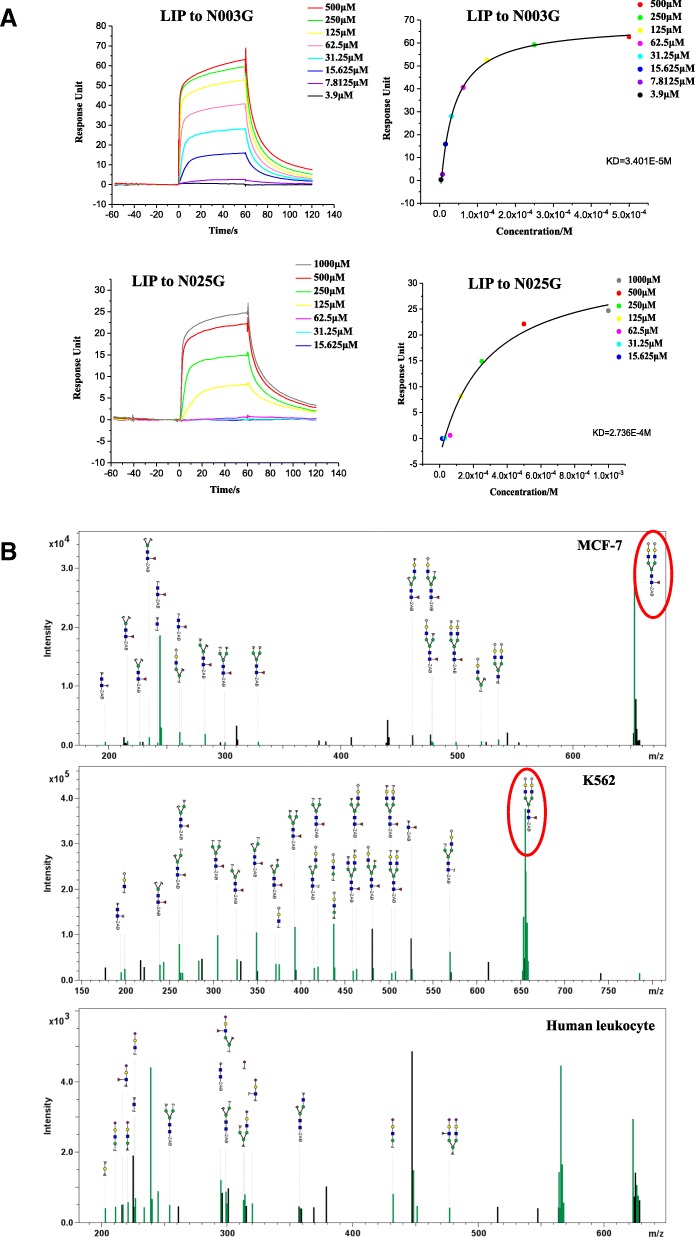
Sialylated antennary N-glycan specificity of LIP. (**a**) BIAcore diagram and saturation curve of LIP bound to N003G and N025G. LIP binds to N003G and N025G with similar low affinities and rapid kinetics. Response units were plotted against protein concentrations. The KD values were calculated by BIAcore T200 analysis software (BIAevaluation version 3.0). (**b**) The MS/MS spectrum of the glycan from the PI-PLC-treated aqueous fraction after Triton X-114 phase separation from MCF-7 and K562 cells and human leukocytes

To identify the specific saccharide that binds to LIP, we applied LIP to a glycan array that contained 100 N-glycan structures on the glycan chip (Additional file 1: Figure S3A). A list of the top two candidate structures with RFUs greater than 10,000 from the screening against 200 μg/mL LIP is shown in Additional file 1: Figure S3B. The results showed that LIP was able to recognize N-glycolylneuraminic acid (Neu5Gc)-containing N-glycans, including the biantennary bisialylated nonfucosylated N-glycan (N003G) and the sialyl Lewis X-containing glycan (N025G). The sialic acid Neu5Gc is present on the sugar chain structures of N025G and N003G and is known to be essential for both SA2,6-Gal and SA2,3-Gal linkages (Additional file 1: Figure S3B). To further characterize the direct interactions between LIP and N025G or N003G, we performed SPR experiments to explore the binding affinity using a BIAcore T200 instrument. The LIP protein was immobilized on a CM5 chip, and N025G or N003G (SugarsTech, China) was then applied to the chip. As expected, LIP exhibited binding to both N025G and N003G; however, the LIP had lower binding affinity for N025G (274 μM) than for N003G (34 μM) (Fig. 4a).

To identify a possible cellular receptor responsible for the rapid loss of cancer cell viability, the glycosidic Neu5Gc of cancer cells was analyzed. According to the method shown in Additional file 1: Figure S3C, the lipid rafts of cancer cells were detected by dot blotting (Additional file 1: Figure S3D), and the interaction of LIP with N-glycan was assessed by incubation with N-glycosidase F. The LIP-bound ingredients were recovered by an injection and recovery program using the BIAcore T200 system. The recovery sample was labeled with 2AB reagent by incubation at 65 °C for 4 h and analyzed by Nano-CaptiveSpray ionization on a QTOF MS instrument (Bruker, Germany) [38]. A glycan search engine (Bruker, Germany) and GlycomeDB (https://glytoucan.org) were used for glycan identification. As expected, the glycan Hex5HexNAc4NeuGc2dHex1, similar to N003G in the N-glycan microarray [39], was found in MCF-7 and K562 cells (Fig. 4b), in contrast to the observation in human normal leukocytes where no glycosidically bound Neu5Gc was found. Humans Neu5Gc is known for its deficiency due to a species-universal inactivating deletion in the CMAH gene encoding the hydroxylase that converts CMP-Neu5Ac to CMP-Neu5Gc. However, Neu5Gc is metabolically incorporated into human tissues from dietary sources (particularly red meat) and is detected at even higher levels in some human cancers [40]. Our studies further confirmed that the MCF-7 and K562 cells contained different levels of Neu5Gc when cultured with fetal bovine serum (FBS) or human serum (Additional file 1: Figure S3E).

### Abrogation of cytocidal activity of LIP against tumor cells by treatment of phosphoinositide phospholipase C (PI-PLC) or sphingomyelinase (SMase)

**Fig. 5:**
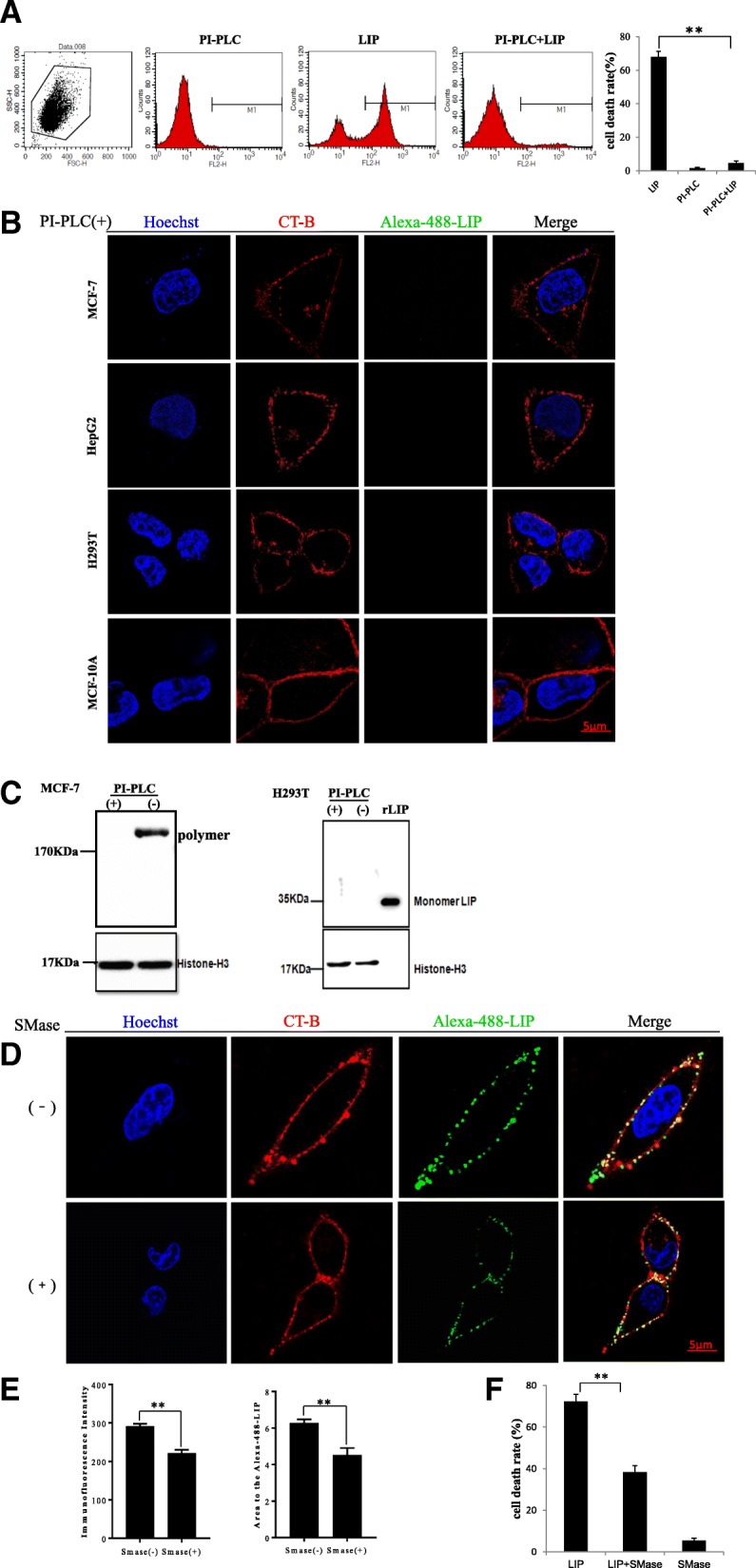
The cytocidal activity of LIP against tumor cells disappeared upon PI-PLC or SMase treatment. (**a**) MCF-7 cells were incubated with (+) or without (−) PI-PLC (5 U/mL) for 2 h and then incubated with LIP and stained with PI for flow cytometric analysis. Histogram showing statistics of the above results (right pane). Means ± SDs are shown (*n* = 3 per group). (**b**) MCF-7, HepG2, H293T and MCF-10A cells were pretreated with PI-PLC and then stained with Alexa555-cholera toxin subunit B (CT-B) prior to staining with Alexa488-tagged LIP. The cells were observed and photographed by 3D-SIM superresolution microscopy. (**c**) MCF-7 cells were incubated with (+) or without (−) PI-PLC prior to incubation with LIP. After the cells were washed to remove free LIP, the proteins were separated by SDS-PAGE and detected by immunoblotting with anti-LIP antibodies (left panel). Immunoblotting of proteins in H293T cells incubated with LIP (right panel). (**d**) MCF-7 cells were pretreated with SMase and then stained with Alexa555-cholera toxin subunit B (CT-B) prior to staining with Alexa488-tagged LIP. (**e**) The mean immunofluorescence intensity, which was measured as the average gray level, and the area ratio of the Alexa488-tagged  LIP area were examined using Image Pro Plus 6.0. (**f**) After the preincubation of MCF-7 cells in the presence (+) or absence (−) of SMase, the cells were treated with LIP. Cell death rates were analyzed by the LDH method. Each histogram represents the average value of triplicate experiments (**P < 0.01). Means ± SDs are shown

Lipid rafts rich in cholesterol, glycolipids, sphingolipids or raft-localizing proteins such as GPI-APs could be candidates for LIP receptors. Previous study showed that PI-PLC could cleave GPI anchor of cell surface [41]. Incubation of PI-PLC pretreated MCF-7 cells with LIP showed a marked reduction in the cell death rate (Fig. 5a). After PI-PLC treatment, Alexa488-tagged LIP was not found on the cell surface of either MCF-7 or HepG2 cells. Interestingly, LIP was not detected on the cell surface of either MCF-10A or H293T cells regardless of PI-PLC treatment (Fig. 5b). In addition, we examined the oligomerization of LIP in PI-PLC-treated and nontreated cells and found that oligomerization occurred in MCF-7 cells not treated with PI-PLC but not in H293T cells regardless of PI-PLC treatment (Fig. 5c). The data showed that PI-PLC appeared to affect the oligomerization efficiency of LIP and the binding process to cancer cells. Furthermore, our results also showed that LIP formed SDS-resistant oligomers, similar to many pore-forming proteins. Taken together, the results suggested that PI-PLC treatment led to removal of the binding receptor of LIP from the cancer cell membrane and hence to the loss of the cell-binding ability, oligomerization and cytocidal activity of LIP.

To gain further insight into the receptors of LIP, we investigated the sphingolipids in lipid rafts. The pretreatment of MCF-7 cells with SMase, which hydrolyzes SM, reduced the binding of LIP to the cell membrane surface (Fig. 5d). Quantification demonstrated that the mean immunofluorescence intensity and the location area of the Alexa-488-LIP were significantly lower in SMase-treated groups (Fig. 5e). In addition, the pretreatment of MCF-7 cells with SMase resulted in a decreased cell death rate after LIP treatment (Fig. 5f). We also investigated whether SM could be cleaved by PI-PLC. Mass spectrometry analysis indicated that the PI-PLC enzyme can cleave SM, as observed with treatment by SMase (Additional file 1: Figure S4A). Hence, PI-PLC was able to cleave both N-linked glycans from GPI-APs and SM on the lipid rafts of cancer cells (Additional file 1: Figure S4B).

### Correlation between SM-binding ability and cytotoxic activity of LIP

**Fig. 6:**
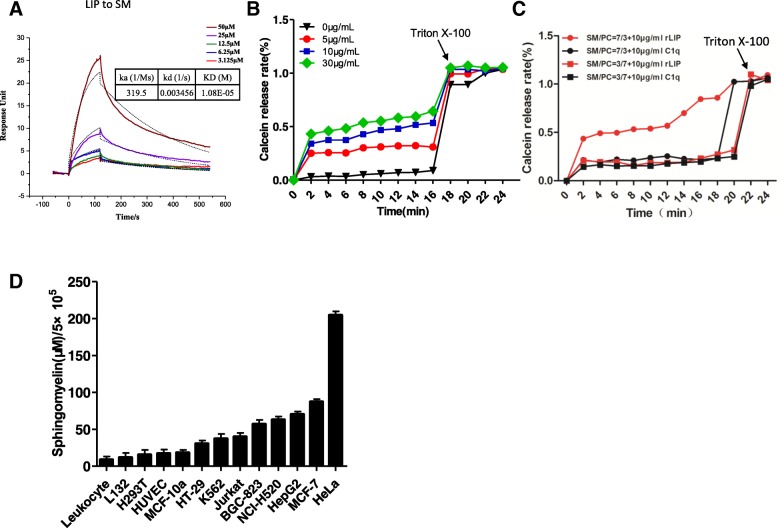
Correlation between the SM-binding ability and cytotoxic activity of LIP. (**a**) BIAcore diagram and saturation curve of LIP bound to SM. LIP binds to SM with similar low affinity and slow kinetics. The KD values were calculated by BIAcore T200 analysis software (BIAevaluation version 3.0). (**b**) Dose-dependent effect of LIP on a liposome membrane composed of a mixture of PC and SM (PC:SM = 1:1). (**c**) The effect of LIP on a liposome membrane composed of a mixture of PC and SM depends on the SM content (PC:SM = 3:7, PC:SM = 7:3). Recombinant L-C1q proteins were used as a negative control in these experiments. (**d**) The content of sphingomyelin in different types of cells

To investigate the possible correlation between LIP and SM, the specific binding of LIP to SM was analyzed by SPR and a liposome lysis assay. In the SPR experiments, LIP was immobilized on a CM5 biosensor surface, over which soluble SM was then injected. The binding affinity of LIP with SM was determined as a KD of 10.8 μM (Fig. 6a). In the liposome lysis assay, when liposomes containing SM were incubated with LIP at 37 °C for 30 min, the release of the entrapped marker (calcein) from liposomes occurred in a dose-dependent manner (Fig. 6b and c). The leakage of liposomes caused by LIP was strictly dependent on the presence of SM and the proportion of SM in the membrane. This relationship implied that LIP perturbed the lipid bilayer structure via the presence of SM in the membranes. However, when a liposome membrane composed of a mixture of PC and CHL containing calcein fluorescence dye was incubated with LIP, the fluorescence intensity was the same with or without LIP treatment [26]. Indeed, the SM content of the normal cell surface was low, which correlated with the resistance to LIP exposure, whereas the SM abundance of cancer cell surfaces could be the major factor leading to increased cell death after LIP exposure (Fig. 6d).

SM synthase (*SMS1/2*) is an enzyme that catalyzes a chemical reaction between two substrates, namely, ceramide and phosphatidylcholine, giving rise to SM and 1,2-diacyl-sn-glycerol. Sphingosine kinase 1 (*SPHK1*) is an enzyme encoded by the *SPHK1* gene and catalyzes the phosphorylation of sphingosine. We investigated the transcription profiles of the *SMS1*, *SMS2* and *SPHK1* genes by real-time PCR. Our data revealed higher expression of SMS1 and SMS2 mRNA in tumor cells and cancer tissues than in normal cells. Similarly, SPHK1 mRNA exhibited higher expression in tumor cells and tissues than in normal cells (Additional file 1: Figure S5).

### Mechanism of the selective recognition cancer cells

**Fig. 7:**
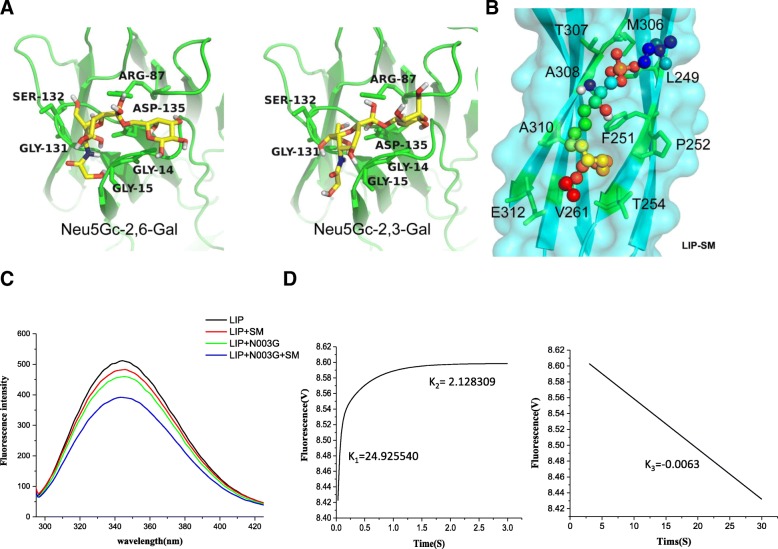
Dual recognition mode of LIP for cancer cells. (**a**) Binding mode of LIP with the disaccharides of Neu5Gc coupled with 2,6-galactose and 2,3-galactose at the N-terminal domain. The disaccharide and the key residues that interacted with it are shown in sticks and colored yellow and green, respectively. The LIP protein is shown in a cartoon representation in green. (**b**) Binding mode of LIP with SM at the C-terminal module. SM is shown as rainbow spheres. LIP protein is shown in cartoon and surface representations and colored cyan. The key residues that interacted with SM are labeled, shown in stick representation and colored green. (**c**) Fluorescence spectra of LIP under different conditions. The LIP stock solutions and N003G or SM stock solutions, respectively, were mixed in phosphate buffer. The resultant mixture was equilibrated for 2 min before recording the steady-state fluorescence spectrum, and the emission spectra were obtained at wavelengths ranging from 290 to 495 nm. Values are the means of five independent experiments. (**d**) Rapid kinetics of the binding of N003G to LIP. Stopped-flow fluorescence measurements of the binding of N003G to LIP. The experiment was performed in PBS at 25 °C. All data sets were analyzed simultaneously with proper weighting to yield best-fit parameters. K_1_ = 24.925540 s^− 1^, K_2_ = 2.128309 ± 0.055980 s^− 1^, K_3_ = -0.0063 s^− 1^

To assess the binding specificity of N-linked glycans and SM for LIP, we estimated the binding free energies by molecular dynamics (MD) simulation and the MM/PBSA method (Additional file 2: Table S5). In brief, we used the FTSite tool to detect putative ligand-binding sites in LIP in both the N-terminal lectin module and C-terminal aerolysin module. The results showed that LIP bound strongly with the disaccharides of Neu5Gc coupled with galactose. Notably, the binding affinity of LIP for the disaccharide of Neu5Gc coupled with 2,6-galactose was much higher than the affinity for Neu5Gc coupled with 2,3-galactose, as evidenced by the energy difference of more than − 46.6 kJ/mol (Fig. 7a), which is consistent with the results of the glycan chip assay experiments. Interestingly, we found that the binding strength of LIP with SM was ~ 3-fold stronger than that with the disaccharides (Fig. 7b), supporting the observation that the K_D_ of LIP-SM interaction (10 μM) was lower than that of LIP-disaccharide (~ 30–50 μM). It must be noted that the binding site of LIP for SM is homologous to that of lysenin, implying that the binding mode of PFPs with SM is conserved among different species.

Next, we wanted to delineate the detailed molecular recognition process in the events of selective killing of tumor cells by LIP. We showed that the interaction of LIP with sugar chains and SM was apparent (Fig. 4 and Fig. 6), supported by the results of the fluorescence spectroscopy assay, in which that the protein fluorescence intensity decreased when N003G and SM were added to the LIP solution. N003G synergized with SM to decrease the fluorescence intensity (Fig. 7c). To investigate whether the formation of LIP was affected by the presence of N003G, the kinetic intermediate of LIP was monitored by measuring the change in fluorescence using a stopped-flow apparatus. As shown in Fig. 7d, three kinetic phases could be observed: two major fast phases, which were completed within 0.03–3 s after the mixing of the reaction in the stopped-flow apparatus, and a minor slow phase, which occurred at a time scale ranging from 3 to 30 s. These results suggested that the reaction between LIP and N003G was swift. Considering all the results, we conclude that the binding of the LIP lectin module with N-linked glycans in the GPI-AP may trigger substantial conformational changes of the aerolysin module, which interacts with SM, ultimately resulting in the formation of a membrane-bound pore in lipid rafts.

### Mutation study of amino acids responsible for killing cancer cells

**Fig. 8:**
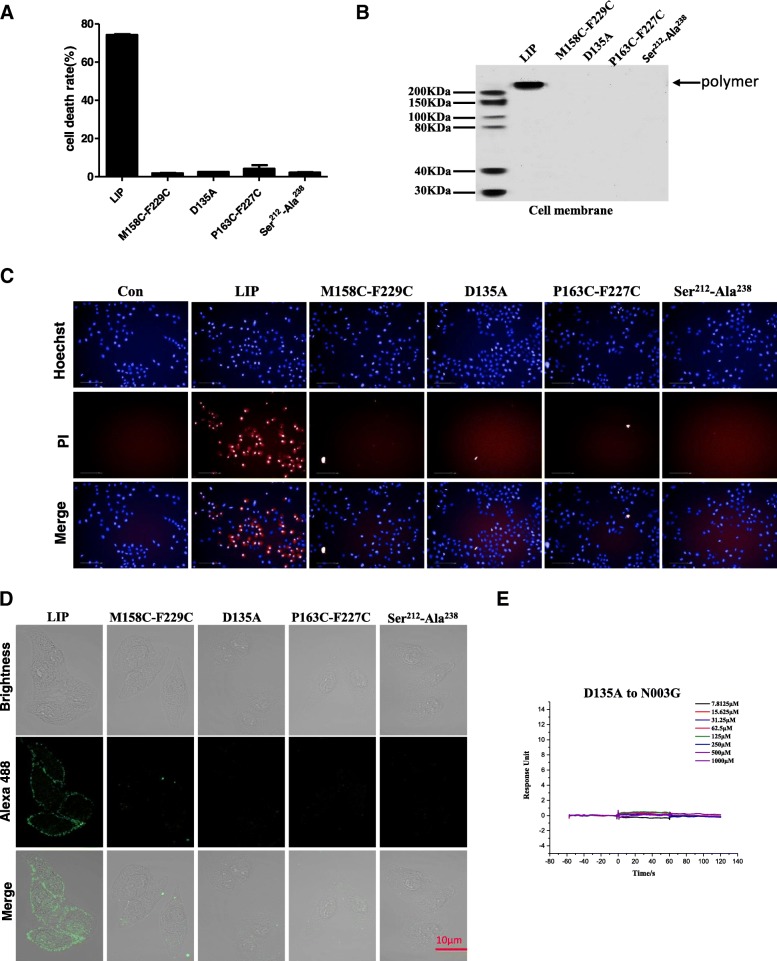
Mutagenesis confirms the key residues. (**a**) MCF-7 cells were treated with LIP or other mutants. MCF-7 cells were plated in 96-well plates at a density of 5 × 10^4^ cells/well and treated with 1 μg/mL LIP and mutants at 37 °C for 24 h. Cell death rates were analyzed by the LDH method. Each histogram represents the average value of triplicate experiments (**P < 0.01). Means ± SDs are shown. (**b**) Immunoblot analysis of LIP and mutants after incubation with MCF-7 cells. The observed bands represent the cell-bound protein from cell membranes. The band corresponding to polymer is indicated with black arrows. (**c**) Staining dead cells with propidium iodide (PI) for high content screening (magnification: 40×). MCF-7 cells were plated in 96-well plates at a density of 5 × 10^4^ cells/well and treated with 1 μg/mL LIP and mutants for 24 h. Cells were washed twice with phosphate-buffered saline (PBS) and stained with PI and Hoechst (Sigma) for 20 min to visualize the cell nuclei. The samples were analyzed on a High Content Screen (PerkinElmer, USA). (**d**) Binding of Alexa488-labeled LIP and mutants to MCF-7 cells. (**e**) BIAcore diagrams of the binding of the D135A mutant of LIP to N003G. The D135A mutant abolished the binding to N003G

According to the LIP structure and the comparison with homologous proteins, it was believed that pre-stem hairpin containing residues 212–238, was involved in the pore-forming process. During the pore-forming process, it was embedded with lipid bilayer and constructed a transmembrane β-barrel. Thus, we performed mutation, such as P163C-F227C, M158C-F229C and deletion of prestem hairpin fragments termed Ser^212^-Ala^238^. Moreover, we also mutated the sugar-binding residue Asp135 to Ala, resulting in mutants termed D135A for short. In contrast to the wild type, the P163C-F227C, M158C-F229C, Ser^212^-Ala^238^, and D135A mutants did not exhibit cytocidal activity against MCF-7 cells (Fig. 8a, c). However, the mutant proteins can be expressed stably and purified (Additional file 1: Figure S6). Our data suggested that the prestem hairpin was fixed to the adjacent core structural domain or directly removed, and a cross-membrane β folding bucket could not be formed. Therefore, it was directly proven that pre-stem hairpin was a necessary component to construct the cross-membrane structural domain. Moreover, the mutants influence oligomerization (Fig. 8b). Consistently, Alexa488-labeled protein mutants could not bind and localize on the membrane surface of MCF-7 cells (Fig. 8d). To further clarify the interactions between LIP and N003G, we performed D135A mutagenesis with N003G and used the SPR experiment to test the binding. The structural analyses in the above section revealed that the Asp135 residues play the major role, contributing most of the interactions. As expected, the Asp135 mutation abolishes the binding to N003G (Fig. 8e). Taken together, the mutagenesis results indicate that the prestem hairpin and ligand-binding loop contribute strongly to cell-binding ability, oligomerization and cytocidal activity against tumor cells.

## Discussion

The ultimate goal of cancer treatment is to find a therapeutic agent (compounds, proteins, peptides or viruses) that as a ‘magic bullet’ by selectively killing cancer cells without side effects on normal cells. The present study confirmed that LIP exhibits interesting features, including the selective recognition and efficient binding of membrane structures of tumor cells and cytocidal activity. Moreover, LIP, as a selective oncolytic agent, can kill a wide range of cancer cell lines without inducing toxicity in primary normal cells.

Over the past decade, emerging evidence suggests that a variety of GPI-APs play fundamental roles in the pathogenesis of a range of cancers [18]. Aerolysin and α-toxin bind to GPI-APs, while cell lines that lack GPI-APs are less sensitive to α-toxin [21]. It has been shown that the glycan region of GPI-APs is involved in aerolysin binding, and the polypeptide moiety is particularly important for toxin-receptor interactions [22]. Here, we found that the binding of LIP with cancer cells was inhibited when treated with PI-PLC, leading to the loss of cytocidal effect of LIP on tumor cells. Moreover, analysis by glycan array identified biantennary bisialylated nonfucosylated N-glycan and sialyl Lewis X-containing glycan structures as the targets recognized by LIP. Importantly, the terminus of the sialylated antennary N-glycan was determined to be Neu5Gc but not Neu5Ac (Additional file 1: Figure S3). In addition, LIP could bind to both SA2,6 Gal and SA2,3 Gal linkages. Cancer cells such as MCF-7 and K562 cells, but not normal cells, possess biantennary bisialylated nonfucosylated N-glycan, which explains the selective killing of LIP. Neu5Gc is an abundant sialic acid in most deuterostome animals but not humans, where the gene encoding CMP-Neu5Ac hydroxylase is inactivated. However, Neu5Gc is still detectable on the surface of human epithelia and endothelia and is highly abundant in tumor cells and malignant tissues [42–44]. In Neu5Gc-deficient animals, a combination of dietary Neu5Gc and circulating anti-Neu5Gc antibodies enhances carcinoma growth [44]. Neu5Gc has also been reported to play an important role in IAV infection in horses and ducks [45]. Sialic acids occupy exposed terminal positions on the oligosaccharide chains of glycoconjugates and serve as ligands for receptors such as selectins and siglecs, which mediate a variety of cell-cell adhesion processes in inflammation and other immune responses [46]. In the present study, more LIP was found on the surface of cancer cells than on normal cells, which correlates with the cell death rate (Fig. 2 and Additional file 1:Figure S1), suggesting that LIP glycosidically bound Neu5Gc on GPI-APs to exert effective cytocidal activity against tumor cells. It has been well documented that PFPs recognize target cells by binding to specific receptors such as sugars, lipids or proteins [25]. Changes in sugar chain structures on the cell surface are known to be associated with the occurrence of cancer and cancer development, invasion and metastasis.

The specific binding of LIP to SM was confirmed by SPR and liposome lysis assays. Interactions of LIP with SM have been documented in actinoporins from sea anemones, where the SM headgroup plays a key role [47, 48]. Lysenin, an SM-dependent PFT from the earthworm *E. fetida*, can interact specifically with SM to confer innate immunity against parasites by attacking them via the formation of pores on the cell membrane [11]. Our study demonstrates for the first time that the PI-PLC enzyme cleaves both GPI-APs and SM in lipid rafts, blocking LIP from binding on the target cell surface and diminishing the cytotoxic activity of LIP. This dual recognition mode of LIP could be important for the selective recognition of cancer cells and the efficacy of its cytocidal activity. Our previous study confirmed that LIP induced the exposure of phosphatidylserine, then bound phosphatidylserine, and finally increased the disruption of cell membrane structure [26]. We believe that LIP protein binds phosphatidylserine in a nonspecific manner, while dual recognition of GPI-APs and SM is a specific and selective recognition mechanism.

Here, the formation of LIP-SM, LIP-N003G and LIP-N003G-SM complexes was studied by monitoring the decrease in fluorescence intensity. As shown in Fig. 7c, the fluorescence intensity of the LIP-N003G-SM complex was lower than the fluorescence intensities of the LIP-N003G or LIP-SM complexes. Equilibrium studies regarding the complexation process have been frequently conducted, but the kinetic aspect of the process has been mostly ignored or not studied due to complications involved in the process. Additionally, the rapid nature of the process makes this process difficult to study by conventional mixing methods [49]. Our kinetic studies, based on a stopped-flow fluorescence method, showed a three-step process of LIP-N003G complexation. As shown in Fig. 7d, three kinetic phases were observed: two major fast phases, which were completed within 0.03–3 s after mixing, and a minor slow phase, which occurred on a time scale ranging from 3 to 30 s. As shown in Fig. 7d, the first and second reaction rates were K_1_ = 24.92554 and K_2_ = 2.128309, respectively, exhibiting increasing and smooth trends, respectively. These results suggested that the first kinetic intermediate might be affected by the presence of N003G, while the second kinetic intermediate might be affected by the presence of SM. We hypothesized that the combination of LIP with N003G/SM leads to a change in LIP structure, exposing aromatic amino acids and decreasing the fluorescence intensity. The BIAcore results also showed that the binding of LIP to N003G occurred faster than that to SM. The current findings indicate that LIP possesses dual recognition sites for both glycosidically bound N003G and SM. However, further investigation is necessary to determine the exact content and sugar type of Neu5Gc in cancer tissues.

Pore formation is commonly exploited by the host as a strategy to mediate physiological processes such as immune defense or development [29]. PFTs and MACPFs share common features and interact with hydrophobic membrane bilayers to form pores. Here, we report the first crystal structure of a lamprey aerolysin-like protein, LIP, revealing that it possesses a β-prism jacalin-like module and an aerolysin module. Sequence alignment and hydrophobicity analysis suggested that a segment of LIP (Ser^212^ to Ala^238^) could form two amphipathic β-strands containing hydrophobic-hydrophilic residues, which are characteristics of membrane-spanning β-hairpins (Fig. 3e-f). Interestingly, the prestem hairpin of LIP shares sequence similarity with the aerolysin family members. The binding components of LIP exhibit a similar structural organization to that of β-PFTs and, notably, contain an amphipathic flexible loop that forms a β-hairpin important for pore formation. These findings suggest that the binding components of LIP and β-PFTs have evolved from a common ancestor.

Jacalin belongs to a family of galactose-binding lectins that contain the jacalin-like lectin domain [50]. However, the jacalin-like lectin domain of Dln1 from *Danio rerio* can specifically bind to high-mannose glycans [13]. The structures of the lectin modules of LIP and Dln1 were superimposed to understand the recognition mechanisms of LIP and Dln1. Indeed, there are major differences, e.g., the groove extending from the binding site of LIP is narrower and deeper than that in Dln1, although the ligand-binding sites are almost identical. In addition, we found that the recognition of sugar chains on cancer cells by LIP is swift, followed by the recognition of SM on lipid rafts.

In summary, we identified a lamprey protein named LIP with cytocidal activity and selective antitumor activities. Structural analysis combined with biochemical assays and analysis of cytocidal activity allowed us to obtain a comprehensive view of the mechanism of action of LIP during pore formation and of the cytocidal activity of this protein. We discovered the dual recognition mechanism of LIP, which is dependent on binding with both N-linked glycans on GPI-APs and SM in lipid rafts. Our results provide valuable information for future studies to elucidate the functions of LIP and LIP-related proteins in lamprey. The unique antitumor effects of LIP certainly warrant further investigation towards the targeted therapy for human cancer.

## Additional File

Additional File 1: **Figure S1.** Localization of LIP in the lipid raft microdomains of cancer cell membranes. Cell lines and primary leukocyte cells isolated from particular individuals (normal and diseased, isolated from peripheral blood) using Ficoll lymphocyte separation medium at a density of 1.077 g/mL. The cells were incubated with Alexa488-tagged LIP (1 μg/mL) at 37 °C for 30 min and subjected to flow cytometric analysis. Histogram showing statistics of the above results. Means ± SDs are shown (*n* = 3 per group). **Figure S2.** Comparative analysis of mannose-specific jacalin-related lectin (mJRL) family members. (A) Sequence alignment of mJRL family members. The residues in the primary carbohydrate binding site are shaded yellow. The residues in a second potential carbohydrate binding site are shaded cyan. Dln1 is from zebrafish (PDB code 4ZNO) [13]; Heltuba is from *Helianthus tuberosus* (PDB code 1C3K) [51]; GRFT (antiviral lectin griffithsin) is from the red alga Griffithsia sp. (PDB code 3LL0) [52]; Banlec is from banana lectin (PDB code 3MIT) [31]; and ZG16p is from human pancreatic lectin (PDB code 3VY7) [53]. The sequences were aligned with Clustal W [54]. (B) Top view of the superimposed lectin module of several mJRL proteins. The 12-stranded β-sheets are labeled. L1 is the GG loop. L6 is the ligand-binding loop. L4 is the ligand recognition loop. L2 and L3 are the ligand-binding loop and GG loop of the putative second binding site. L5 was also found to be involved in ligand binding in Banlec. LIP is shown in sandy brown. Dln1 is shown in blue (sucrose) and magenta (mannose). GRFT is shown in green. Banlec is shown in salmon. Heltuba is shown in purple. ZG16p is shown in gray. **Figure S3.** Sialylated antennary N-glycan specificity of LIP. (A) 100 N-glycan identification list. The numbers of complex and hybrid NgGlycans, high-mannose N-glycans and Neu5Gc N-glycans are 81, 10 and 9, respectively. (B) Typical binding of LIP assay result from the 100 N-Glycan Array. 103: Biotinylated mannose (0.01 mg/mL), 104: Human IgG (0.01 mg/mL), 105: Mouse IgG (0.1 mg/mL) as a positive control. (C) The N-glycosidase F-treated aqueous fraction from MCF-7, K562 cells and human leukocytes after Triton X-114 phase separation was digested with trypsin and analyzed by MS/MS. The general scheme and chemical treatments used in this study (left pane). (D) Distribution profile of lipid raft components in DRMs by sucrose density gradient centrifugation. Cells were lysed in MBS buffer containing 1% Triton X-100 at 4 °C. Lysates were fractionated by sucrose gradient centrifugation, and 7 fractions were collected from the top of the centrifuge tube. A sample from each fraction was subjected to dot immunoblotting analysis using antibodies to flotillin-I to confirm the fraction of lipid raft components. (E) MCF-7 and K562 cells and human leukocytes were examined with anti-Neu5Gc antibodies in culture containing either fetal bovine serum or human serum. Values are the means of three independent experiments. Means ± SDs are shown. **Figure S4.** The effect of PI-PLC on GPI-APs and SM. (A) MALDI-TOF MS spectra of SM standard treated with PI-PLC and sphingomyelinase (SMase). After treatment with PI-PLC and SMase, the content of the SM standard (m/z = 703.56) decreased. (B) The general scheme of GPI-APs and SM structure in mammalian cells. **Figure S5.** Relative expression of the mRNA of SM-related synthetases in different tumor cells measured by real-time PCR. **Figure S6.** Protein expression and purification. The LIP mutants were constructed using a Site-directed Mutagenesis Kit (Thermo Scientific) and then were expressed, refolded and purified following the same procedures as for wild-type LIP protein. (PDF 1456 kb)

Additional File 2: **Table S1.** Cytocidal activities of LIP against various tumor cells. **Table S2**. The effect of LIP on normal and primary cells. **Table S3.** Data collection and refinement statistics for LIP. One crystal was used for each structure. Values in parentheses are for the highest resolution shell. *Rmerge*=ΣhΣi|*Ih,i*-*Ih*|/ΣhΣi*Ih,i*, where *Ih* is the mean intensity of the *i* observations of symmetry related reflections of *h*. *R*=Σ|*Fobs*-*Fcalc*|/Σ*Fobs*, where *Fcalc* is the calculated protein structure factor from the atomic model (Rfree was calculated with 5% of the reflections selected randomly). **Table S4.** Data of Z scores and RMSDs for each PDB comparison. **Table S5.** Binding free energy calculation by MM/PBSA method after molecular dynamics (MD) simulation by Gromacs. ∆E_vdw_: van der Waal energy; ∆E_ele_: electrostatic energy; ∆G_PB_: polar salvation energy; ∆G_SA_: non-polar salvation energy;∆G_binding_: binding energy. (DOCX 28 kb)

## Abbreviations

DOPC: Dioleoylphosphatidylcholine
GPI-APs: GPI-anchored proteins
MAC: Membrane attack complex
PFPs: Pore-forming proteins
PFTs: Pore-forming toxins
PI-PLC: Phosphoinositide phospholipase
RFUs: Relative fluorescence units
SM: Sphingomyelin
SMase: Sphingomyelinase
