# Supplementary figures and images for: Crystal structure of a cytocidal protein from lamprey and its mechanism of action in the selective killing of cancer cells
Source: Cell Commun Signal. 2019 May 27;17:54. doi: 10.1186/s12964-019-0358-y (PMC6537362; doi:10.1186/s12964-019-0358-y)

Fig. S1

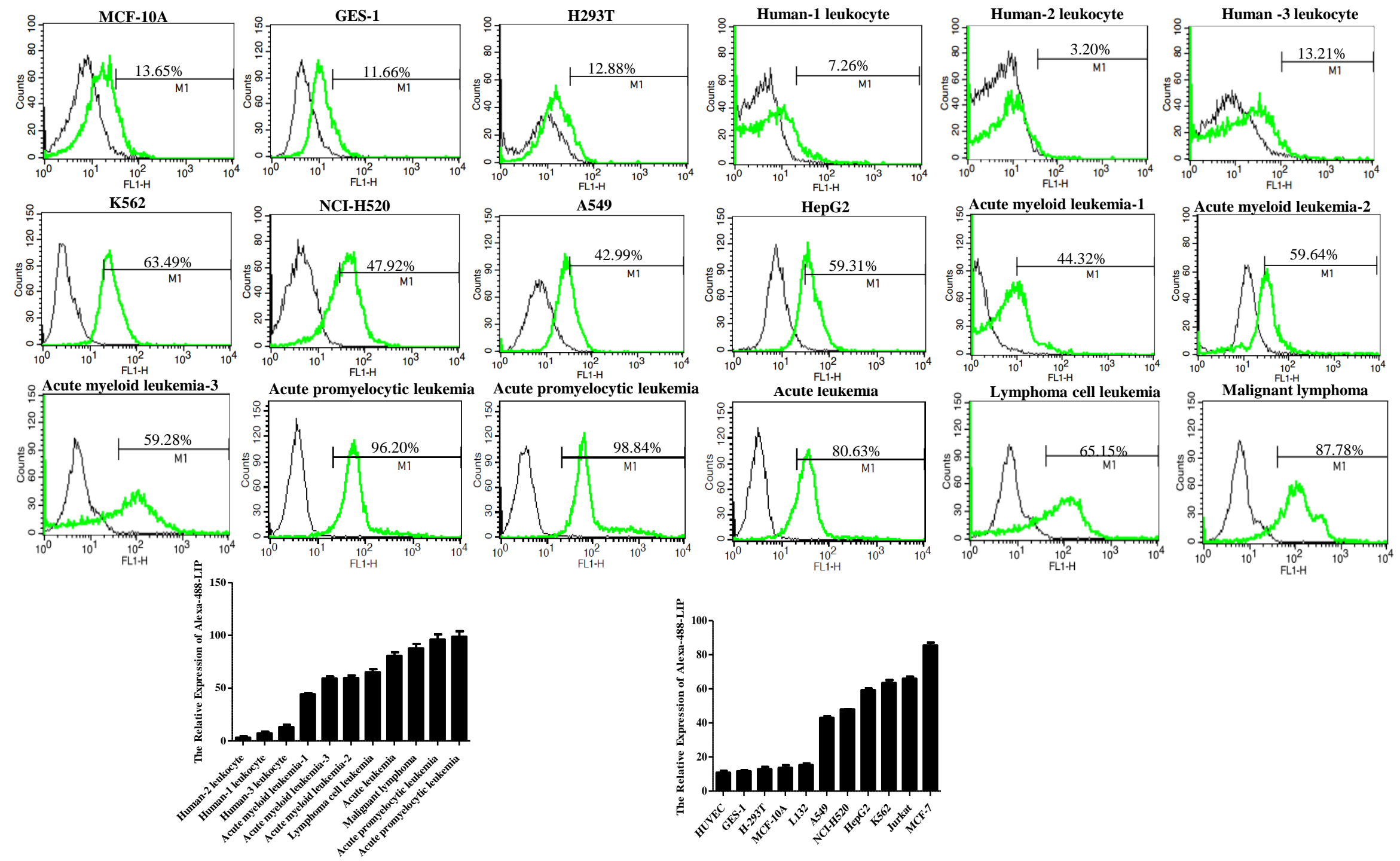

A

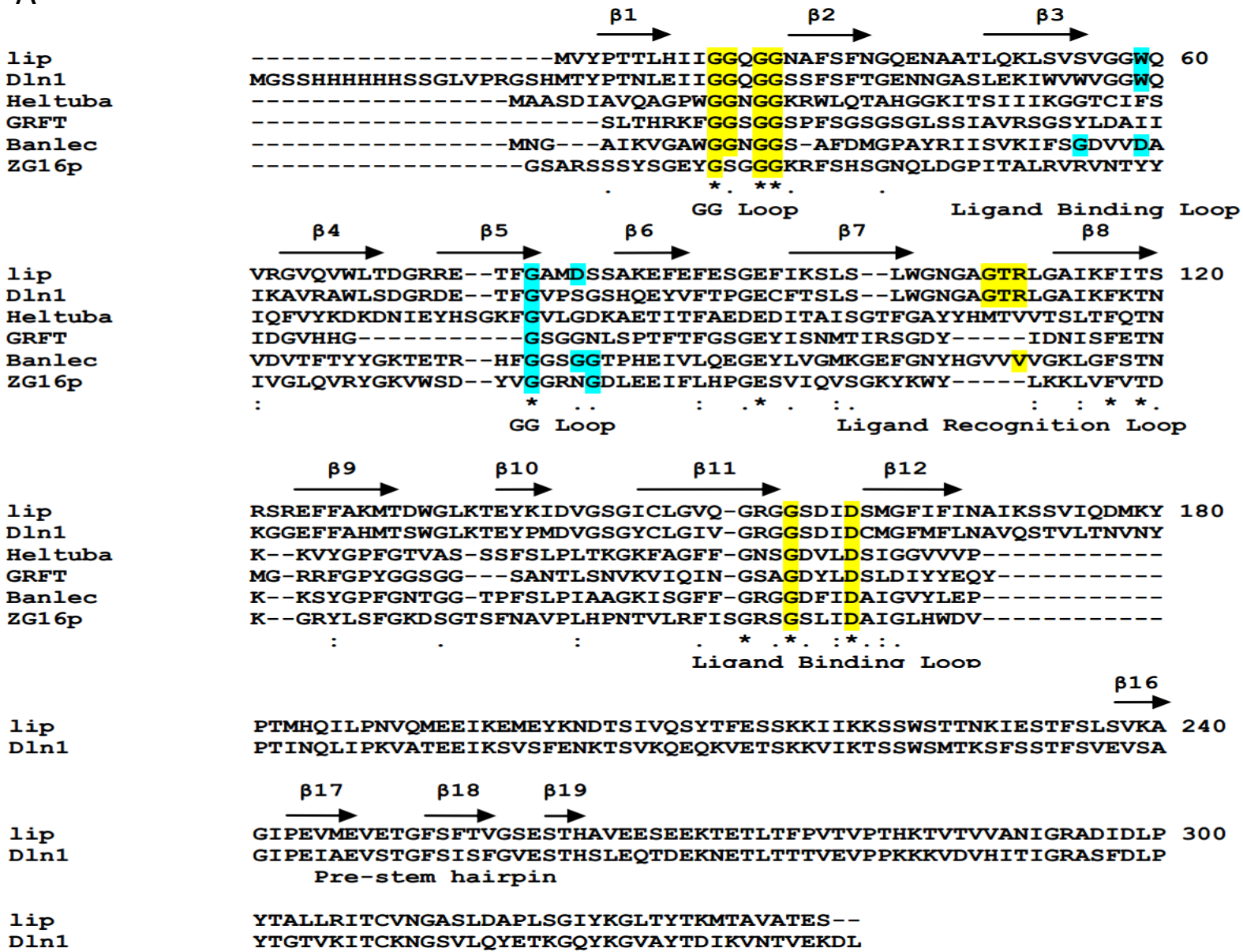

B

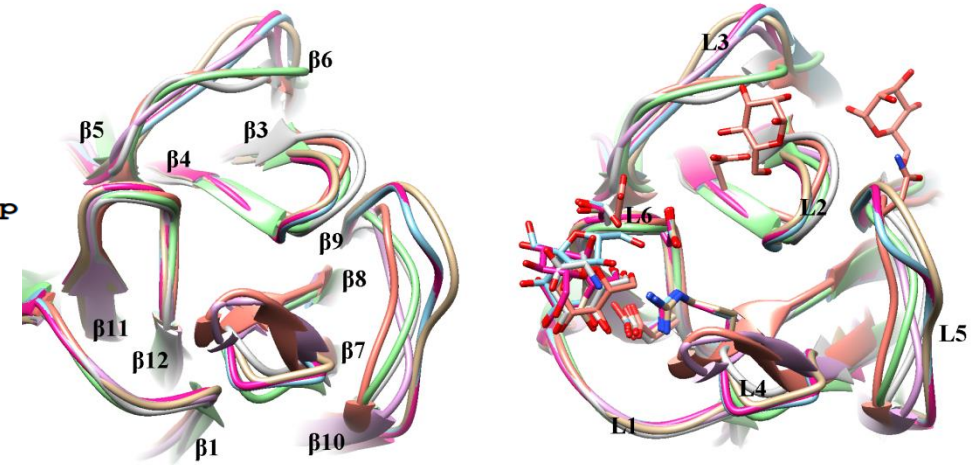

# A Complex and Hybrid N-Glycans

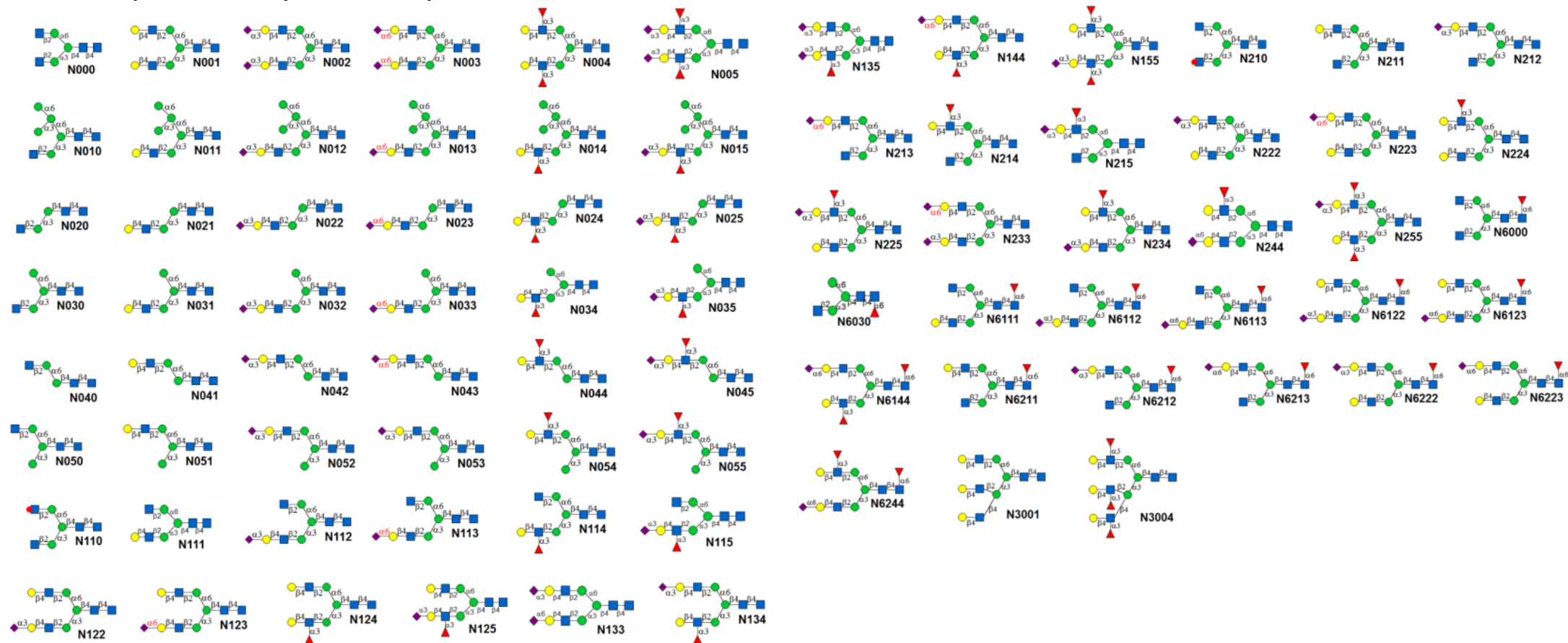

## High-Mannose N-Glycans

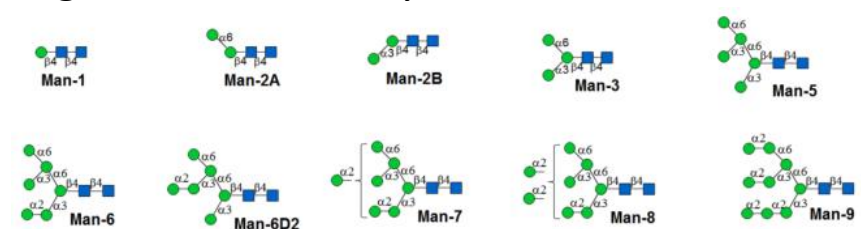

## Neu5Gc N-Glycans

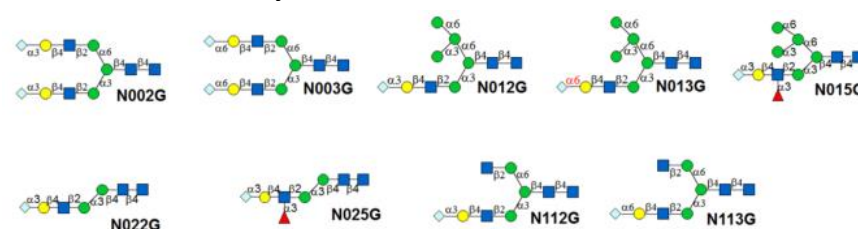

B

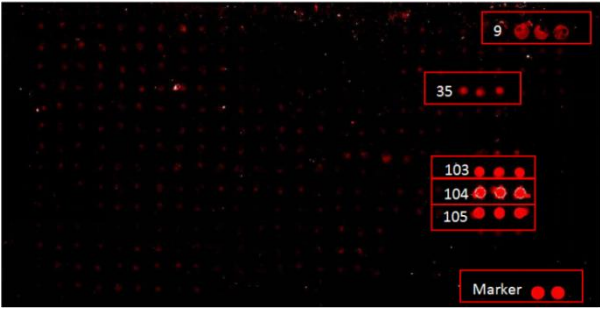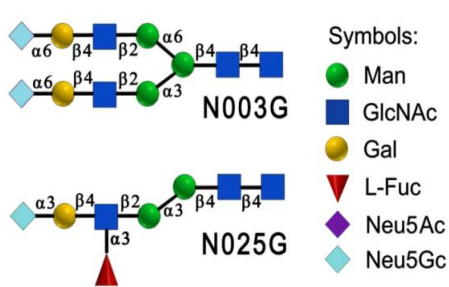

D

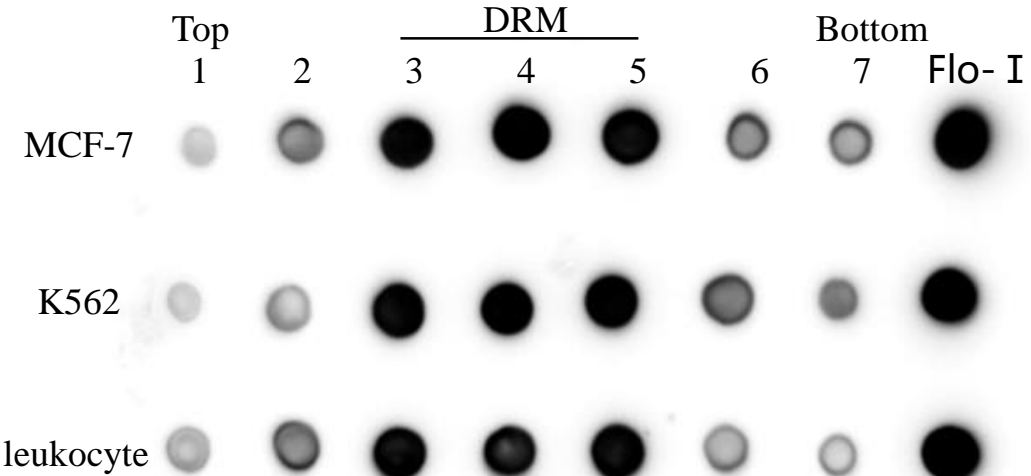

C

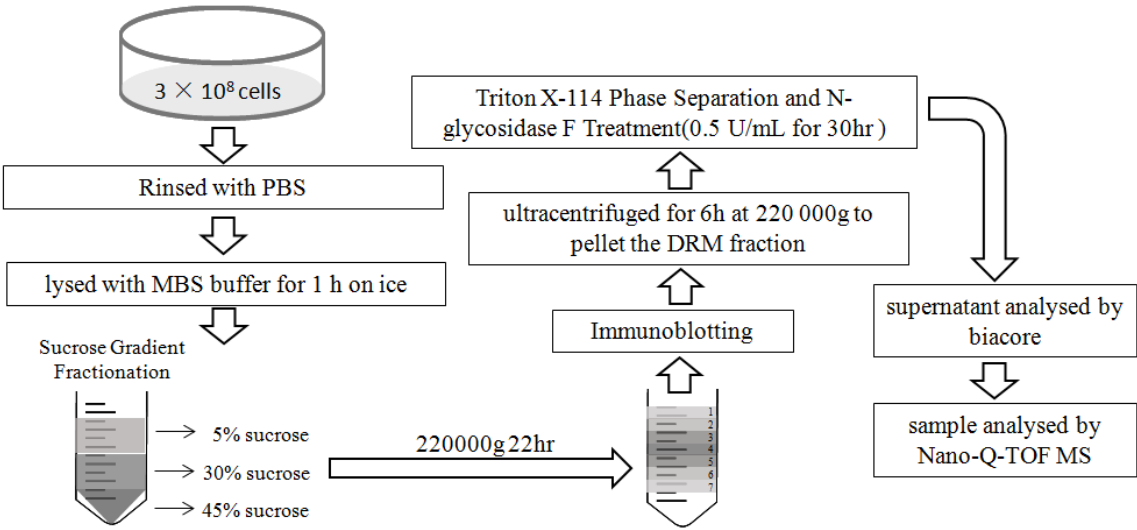

E

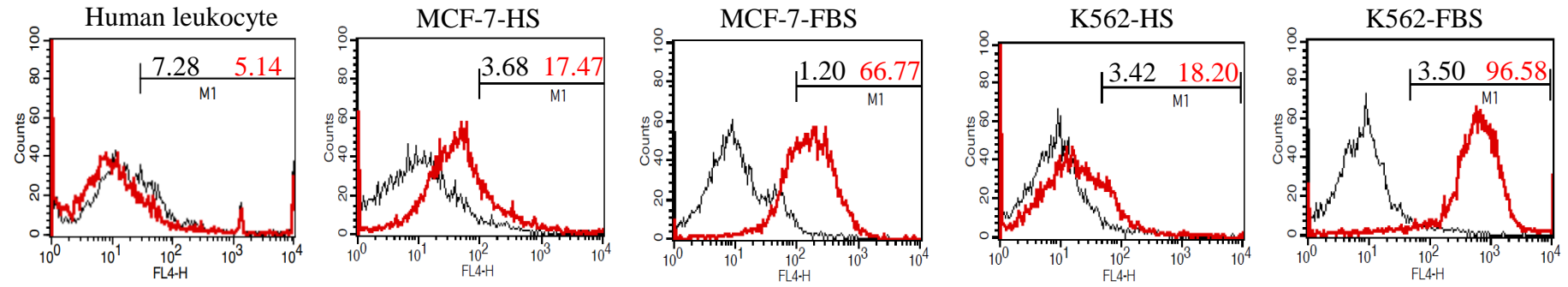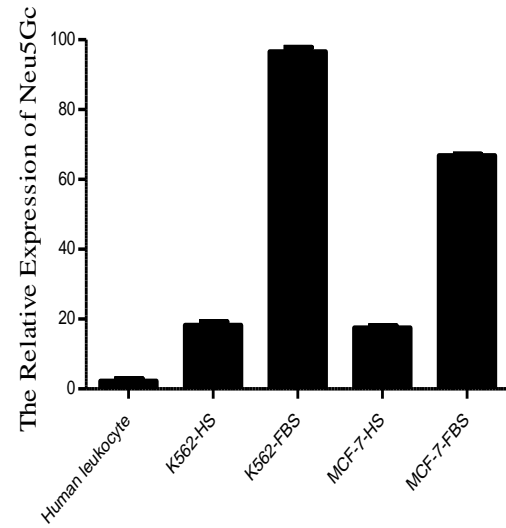

A

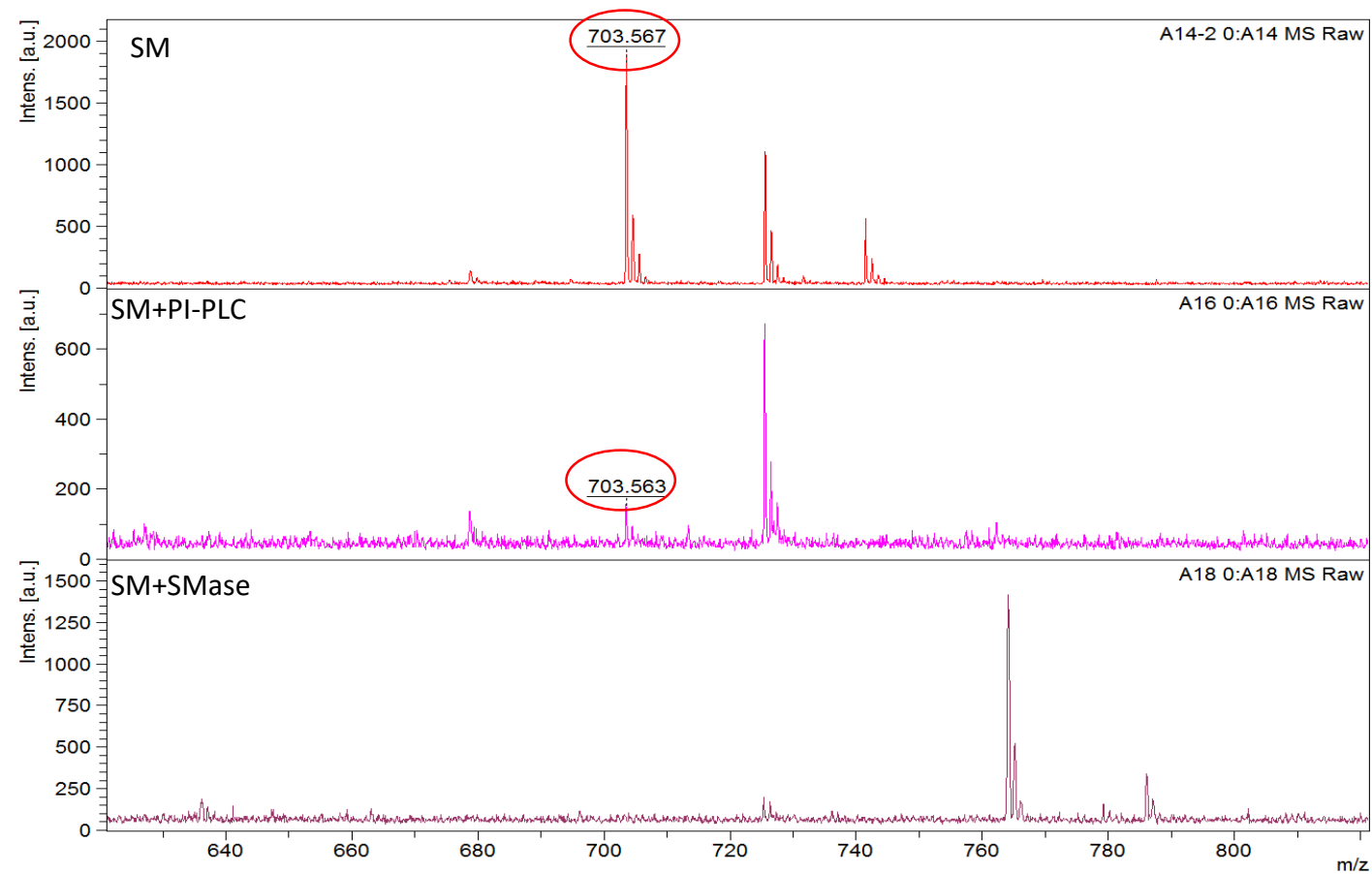

B

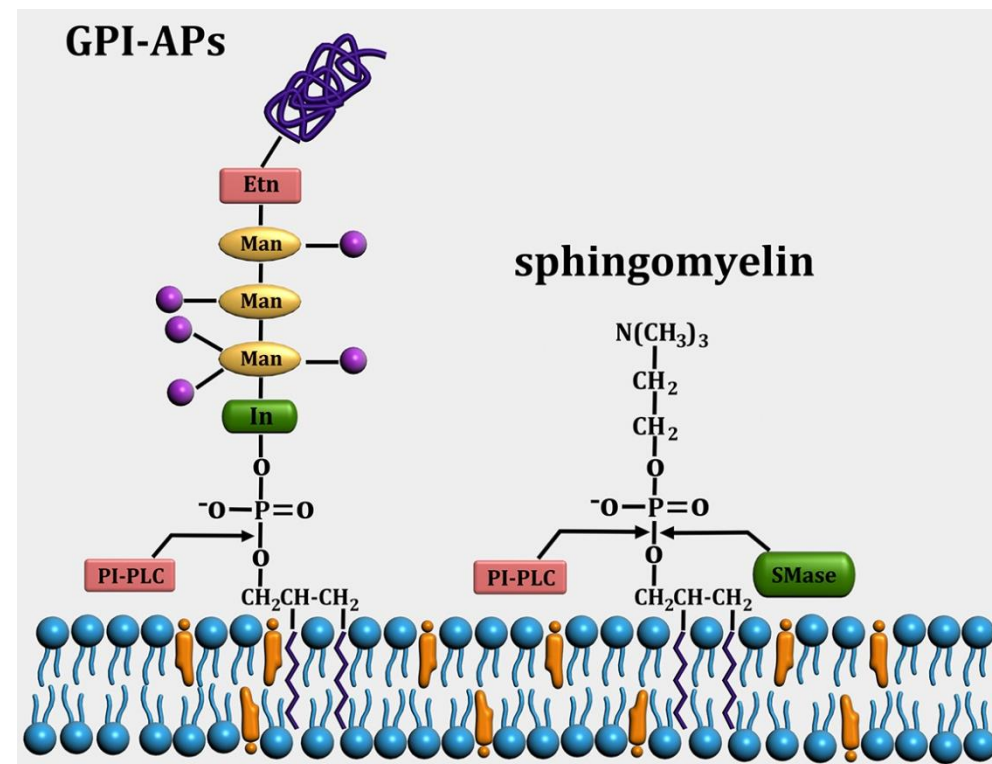

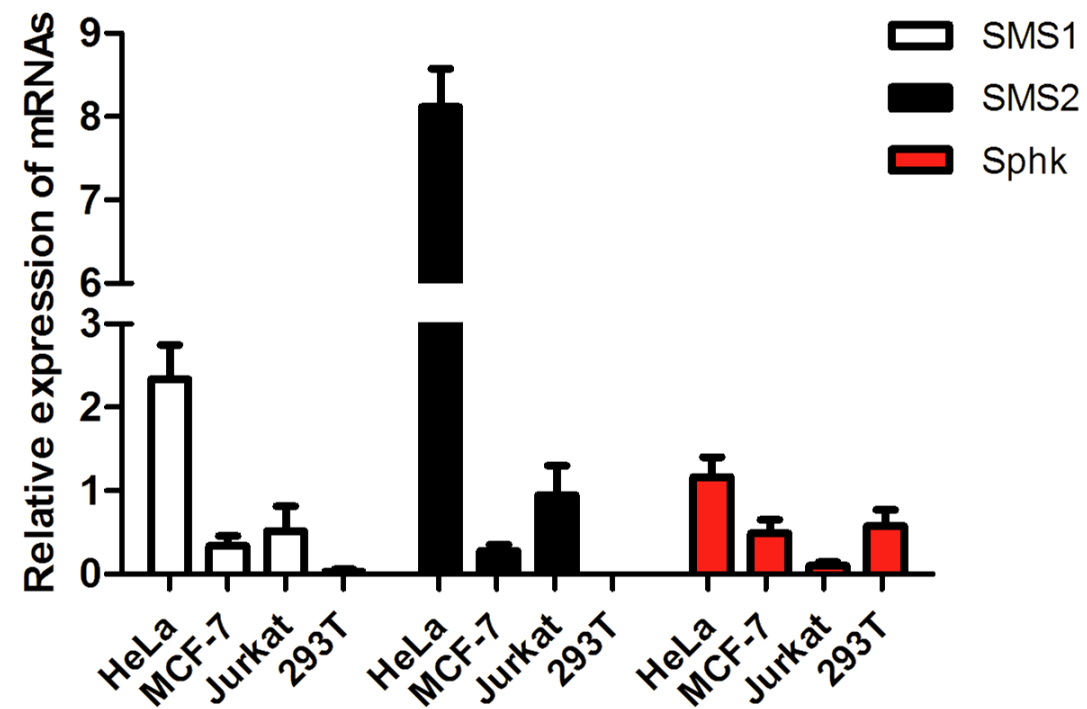

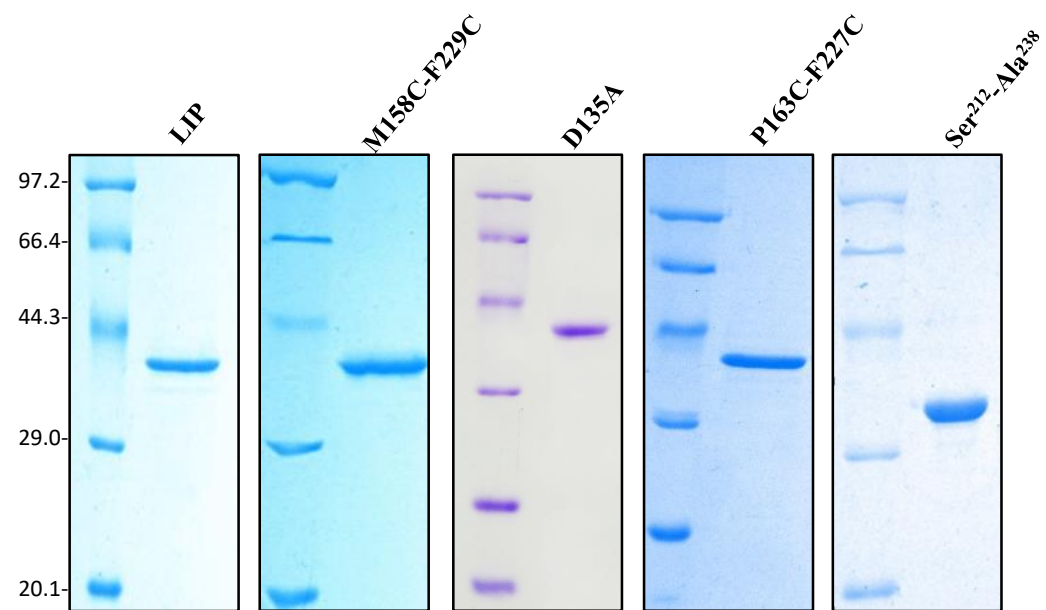

Supplement: Supplementary file 1 — Figure S1. Localization of LIP in the lipid raft microdomains of cancer cell membranes. Cell lines and primary leukocyte cells isolated from particular individuals (normal and diseased, isolated from peripheral blood) using Ficoll lymphocyte separation medium at a density of 1.077 g/mL. The cells were incubated with Alexa488-tagged LIP (1 μg/mL) at 37 °C for 30 min and subjected to flow cytometric analysis. Histogram showing statistics of the above results. Means ± SDs are shown (n = 3 per group). Figure S2. Comparative analysis of mannose-specific jacalin-related lectin (mJRL) family members. (A) Sequence alignment of mJRL family members. The residues in the primary carbohydrate binding site are shaded yellow. The residues in a second potential carbohydrate binding site are shaded cyan. Dln1 is from zebrafish (PDB code 4ZNO) [13]; Heltuba is from Helianthus tuberosus (PDB code 1C3K) [51]; GRFT (antiviral lectin griffithsin) is from the red alga Griffithsia sp. (PDB code 3LL0) [52]; Banlec is from banana lectin (PDB code 3MIT) [31]; and ZG16p is from human pancreatic lectin (PDB code 3VY7) [53]. The sequences were aligned with Clustal W [54]. (B) Top view of the superimposed lectin module of several mJRL proteins. The 12-stranded β-sheets are labeled. L1 is the GG loop. L6 is the ligand-binding loop. L4 is the ligand recognition loop. L2 and L3 are the ligand-binding loop and GG loop of the putative second binding site. L5 was also found to be involved in ligand binding in Banlec. LIP is shown in sandy brown. Dln1 is shown in blue (sucrose) and magenta (mannose). GRFT is shown in green. Banlec is shown in salmon. Heltuba is shown in purple. ZG16p is shown in gray. Figure S3. Sialylated antennary N-glycan specificity of LIP. (A) 100 N-glycan identification list. The numbers of complex and hybrid NgGlycans, high-mannose N-glycans and Neu5Gc N-glycans are 81, 10 and 9, respectively. (B) Typical binding of LIP assay result from the 100 N-Glycan Array. 103: Biotinylated [file 12964_2019_358_MOESM1_ESM.pdf]
