# Supplementary material for: Crystal structure of a cytocidal protein from lamprey and its mechanism of action in the selective killing of cancer cells
Source: Cell Commun Signal. 2019 May 27;17:54. doi: 10.1186/s12964-019-0358-y (PMC6537362; doi:10.1186/s12964-019-0358-y)
Supplement: Supplementary file 2 — Table S1. Cytocidal activities of LIP against various tumor cells. Table S2. The effect of LIP on normal and primary cells. Table S3. Data collection and refinement statistics for LIP. One crystal was used for each structure. Values in parentheses are for the highest resolution shell. Rmerge=ΣhΣi|Ih,i-Ih|/ΣhΣiIh,i, where Ih is the mean intensity of the i observations of symmetry related reflections of h. R=Σ|Fobs-Fcalc|/ΣFobs, where Fcalc is the calculated protein structure factor from the atomic model (Rfree was calculated with 5% of the reflections selected randomly). Table S4. Data of Z scores and RMSDs for each PDB comparison. Table S5. Binding free energy calculation by MM/PBSA method after molecular dynamics (MD) simulation by Gromacs. ∆Evdw: van der Waal energy; ∆Eele: electrostatic energy; ∆GPB: polar salvation energy; ∆GSA: non-polar salvation energy;∆Gbinding: binding energy. (DOCX 28 kb) [file 12964_2019_358_MOESM2_ESM.docx]

**Table S1. Cytocidal activities of LIP against various tumor cells**

| **Cell line** | **Disease** | **LD50(μg/mL)** | **Cell line** | **Disease** | **LD50(μg/mL)** |
| --- | --- | --- | --- | --- | --- |
| A549 | Adenocarcinoma | 9.20 | K562 | Chronic myeloid leukemia | 0.63 |
| NCI-H520 | Squamous cell carcinoma | 5.24 | Jurkat | Leukemia T cells | 0.98 |
| NCI-H446 | Small cell lung cancer | 2.50 | NB4 | Acute promyelocytic | 0.74 |
| A431 | Squamous cell carcinoma | 3.45 | HL-60 | Acute promyelocytic leukemia | 0.80 |
| MDA-MB-231 | Breast adenocarcinoma | 6.21 | Raji | Lymphoma cells | 1.25 |
| MDA-MB-453 | Breast adenocarcinoma | 5.75 | HT-1080 | Human fibrosarcoma cells | 1.50 |
| MDA-MB-468 | Breast adenocarcinoma | 5.55 | SMMC-7721 | Hepatocellular carcinoma | 0.92 |
| T-47D | Mammary ductal carcinoma | 3.72 | HepG2 | Hepatocellular carcinoma | 0.75 |
| MCF-7 | Breast adenocarcinoma | 0.52 | DU 145 | Prostate carcinoma | 3.5 |
| Bcap-37 | Breast medullary carcinoma cell | 2.50 | BGC-823 | Human gastric adenocarcinoma | 3.14 |
| HeLa | Cervix adenocarcinoma | 2.45 | HT-29 | Colon cancer cells | 2.5 |
| SKOV3 | Ovarian cancer cells | 1.36 |  |  |  |

**Table S2.** **The effect of LIP on** **normal and primary cells**

| **Name** | **Tissue** | **Time(h)** | **Dose(μg/mL)** | **Death rate(%)** |
| --- | --- | --- | --- | --- |
| 293T  MCF-10A | Human embryonic kidney cell  Human breast epithelial cell line | 72  72 | 20  20 | 12  12.5 |
| L132 | Human lung epithelial cells | 72 | 20 | 15.8 |
| HUVEC | Human umbilical vein endothelial cells | 72 | 20 | 10 |
| GES-1 | Human gastric mucosal cells | 72 | 20 | 8.9 |
| NIH3T3  CHO-K1  COS-7  Rabbit erythrocyte  Human leukocyte | Mouse embryonic fibroblastsl  Chinese hamster ovarian cancer  Monkey kidney tissue  Blood  Blood | 72  72  72  72  72 | 20  20  20  20  20 | 14.5  10  8.5  15.4  7.8 |

**Table S3. Data collection and refinement statistics for LIP**

| **Crystal Form** | **LIP** |
| --- | --- |
| **Data collection** |  |
| Space Group | P4_3_2_1_2 |
| Cell dimensions |  |
| a, b, c (Å) | 103.26, 103.26,166.21 |
| α, β, γ, (°) | 90, 90, 90 |
| Resolution (Å)  R_merge_ | 44.5~2.25  64.3(14.6) |
| R_meas_  R_pim_  CC1/2 | 66.4(15.1)  16.2(0.037)  93.2(98.8) |
|  |  |
| I / σI | 3.38(28.3) |
| Completeness (%) | 100(99.9) |
| Redundancy | 16.3 (16.0) |
| **Refinement** |  |
| No. reflections | 43222 |
| R_work_ / R_free_ (%) | 19.8/ 23.2 |
| No. atoms |  |
| Protein | 4820 |
| Wilson B-factors(Å^2^) |  |
| Average B(Å^2^) | 34 |
| R.m.s. deviations |  |
| Bond lengths (Å) | 0.013 |
| Bond angles (°) | 1.947 |
| Ramachandran plot statistics (%) |  |
| Most favoured | 98.1% |
| Additional allowed | 1.9% |
| Generously allowed | 0.0 |
| Disallowed | 0 |
| PDB code | 6IUL |

One crystal was used for each structure. Values in parentheses are for the highest resolution shell. *Rmerge*=ΣhΣi|*Ih,i*-*Ih*|/ΣhΣi*Ih,i*, where *Ih* is the mean intensity of the *i* observations of symmetry related reflections of *h*. *R*=Σ|*Fobs*-*Fcalc*|/Σ*Fobs*, where *Fcalc* is the calculated protein structure factor from the atomic model (Rfree was calculated with 5% of the reflections selected randomly).

**Table S4. Data of Z scores and RMSDs for each PDB comparison**

| N-terminal lectin module | Z | rmsd | lali | nres | %id | PDB Description |
| --- | --- | --- | --- | --- | --- | --- |
| 4zno-B | 28.9 | 0.7 | 144 | 318 | 66 | NATTERIN-LIKE PROTEIN |
| 3vy7-A | 18 | 2.1 | 133 | 139 | 22 | ZYMOGEN GRANULE MEMBRANE PROTEIN 16 |
| 1c3k-A | 17.5 | 2.2 | 132 | 143 | 18 | AGGLUTININ |
| 3mit-A | 17.2 | 2 | 130 | 138 | 22 | LECTIN |
| 3ll0-A | 16.8 | 2.2 | 123 | 125 | 23 | GRIFFITHSIN |
| C-terminal aerolysin module | Z | rmsd | lali | nres | %id | PDB Description |
| 4zno-A | 21.9 | 1.1 | 169 | 318 | 49 | NATTERIN-LIKE PROTEIN |
| 1w3a-A | 12.2 | 3.3 | 155 | 312 | 21 | HEMOLYTIC LECTIN LSLA |
| 1pre-B | 6.7 | 4.7 | 147 | 451 | 15 | PROAEROLYSIN |

**Table S5. Binding free energy calculation by MM/PBSA method after molecular dynamics (MD) simulation by Gromacs**

| **Contribution(kJ/mol)** | **Neu5Gc-alpha-2,6- Gal** | **Neu5Gc-alpha-2,3- Gal** | **SM** |
| --- | --- | --- | --- |
| ∆E_vdw_ | -91.105 ± 0.923 | -55.422 ± 2.008 | -96.715 ± 2.299 |
| ∆E_ele_ | -208.980 ± 2.421 | -155.222 ± 5.145 | -413.576 ± 5.865 |
| ∆G_PB_ | 161.321 ± 1.966 | 114.948 ± 3.141 | 81.307 ± 3.896 |
| ∆G_SA_ | -11.226 ± 0.057 | -7.539 ± 0.148 | -13.040 ± 0.258 |
| ∆G_binding_ | **-149.908 ± 2.441** | **-103.385 ± 2.882** | **-442.103 ± 5.500** |

∆E_vdw_: van der Waal energy; ∆E_ele_: electrostatic energy; ∆G_PB_: polar salvation energy; ∆G_SA_: non-polar salvation energy;

∆G_binding_: binding energy
